# Assembly of Influenza Hemagglutinin Fusion Peptides in a Phospholipid Bilayer by Coarse-grained Computer Simulations

**DOI:** 10.3389/fmolb.2015.00066

**Published:** 2015-11-18

**Authors:** Francesca Collu, Enrico Spiga, Christian D. Lorenz, Franca Fraternali

**Affiliations:** ^1^Randall Division of Cell and Molecular Biophysics, Bioinformatics Computational Biology, King's College LondonLondon, UK; ^2^Mill Hill Laboratory, Mathematical Biology, The Francis Crick InstituteLondon, UK; ^3^Theory and Simulation of Condensed Matter Group, Department of Physics, King's College LondonLondon, UK

**Keywords:** assembly, influenza fusion peptides, molecular dynamics simulations, coarse-grained simulations, phospholipid bilayer, theoretical investigation

## Abstract

Membrane fusion is critical to eukaryotic cellular function and crucial to the entry of enveloped viruses such as influenza and human immunodeficiency virus. Influenza viral entry in the host cell is mediated by a 20–23 amino acid long sequence, called the fusion peptide (FP). Recently, possible structures for the fusion peptide (ranging from an inverted V shaped α-helical structure to an α-helical hairpin, or to a complete α-helix) and their implication in the membrane fusion initiation have been proposed. Despite the large number of studies devoted to the structure of the FP, the mechanism of action of this peptide remains unclear with several mechanisms having been suggested, including the induction of local disorder, promoting membrane curvature, and/or altering local membrane composition. In recent years, several research groups have employed atomistic and/or coarse-grained molecular dynamics (MD) simulations to investigate the matter. In all previous works, the behavior of a single FP monomer was studied, while in this manuscript, we use a simplified model of a tripeptide (TP) monomer of FP (TFP) instead of a single FP monomer because each Influenza Hemagglutinin contains three FP molecules in the biological system. In this manuscript we report findings targeted at understanding the fusogenic properties and the collective behavior of these trimers of FP peptides on a 1-palmitoyl-2-oleoyl-sn-glycero-3-phosphocholine model membrane. Here we show how the TFP monomers self-assemble into differently sized oligomers in the presence of the membrane. We measure the perturbation to the structure of the phospholipid membrane caused by the presence of these TFP oligomers. Our work (i) shows how self-assembly of TFP in the presence of the membrane induces non negligible deformation to the membrane and (ii) could be a useful starting point to stimulate discussion and further work targeted to fusion pore formation.

## 1. Introduction

The Influenza virus is responsible for hundreds of thousands of deaths every year. The situation becomes even worse in pandemic years, when a subtype which was not previously circulating emerges and spreads among the human population. Influenza is, therefore, a major health concern, and the development of effective drugs against this virus is one of the biggest quests of modern medicine. Like all enveloped viruses, it is well established that the surface glycoprotein covering the influenza viral capsid, influenza hemagglutinin (HA), is known to be responsible for binding to cells and the fusion of the viral and endosomal membranes (Wiley and Skehel, 1987; Skehel and Wiley, 2000; Skehel et al., 2001). This fusion process is mediated by the glycoprotein hemagglutinin (HA; Luo, 2012). HA is composed of three identical subunits and the N-terminal end of these subunits contains a sequence of 20 N-terminal amino acids (GLFGAIAGFIENGWEGMIDG) that is called the fusion peptide (FP; Cohen and Melikyan, 2001). During the viral infection process, a decrease in the local pH level provokes an extensive conformational rearrangement in each HA0, which unfurls the HA1 and HA2 chains (Durrer et al., 1996). As part of this unfurling process, the individual FPs are revealed and are inserted into the membrane of the healthy cell (Carr et al., 1997).

The mechanism and structure of the single FP as it inserts into the membrane of the healthy cell have been the focus of several experimental studies. Han and Tamm (2000) have used circular dichroism (CD) spectroscopy and Attenuated Total Reflection (ATR)-Fourier Transform Infrared (FTIR) measurements to show that the FP adopt an α-helical structure upon inserting into model lipid membranes, which have a 4:1 molar ratio of 1-palmitoyl-2-oleoyl-sn-glycero-3-phosphocholine (POPC) to 1-palmitoyl-2-oleoyl-sn-glycero-3-phosphoglycerol (POPG).

By combining the results of electron paramagnetic resonance (EPR), CD and NMR measurements, Han et al. also have proposed that FP inserted into dodecylphosphocholine (DPC) micelles and 4:1 POPC:POPG bilayers adopts an inverted V structure (Han et al., 2001), in which both the N- and C-terminal ends of the peptide are buried deeper into the membrane than the glycerol group and the 12 ASN residue is closest to the lipid membrane interface. Tamm and coworkers proposed that influenza HA-mediated membrane fusion occurs via a spring-loaded boomerang mechanism, where the FP inserts into the target membrane and adopts a boomerang structure (Tamm, 2003). The boomerang structure is characterized by a kink angle within the inverted V structure of ~120° (Tamm, 2003), which is centered around the 12 ASN residue (Tamm et al., 2003; Lai and Tamm, 2007).

This vision has been recently challenged by Bax's group that has found that at low pH, where the fusion process is triggered, the native peptide transiently visits activated states which consist of two stable helices switching between L-shape and extended arrangements (Lorieau et al., 2011a,b, 2012). Bax's group refers to the boomerang structure with the name of hairpin structure. The opening of the hairpin structure has been thought to be essential for the formation of a membrane pore at the final stage of the fusion process (Lorieau et al., 2010, 2013a,b). Recently it has been shown that HA FP functions by inducing membrane curvature (Smrt et al., 2015).

Despite the large number of studies devoted to the study of the FP, the mechanism of action of this peptide remains unclear. On the basis of experimental findings, several mechanisms have been suggested, such as the induction of local disorder, promoting membrane curvature, or altering local membrane composition (Epand, 2003; Chernomordik and Kozlov, 2008). Steady state fluorescence experiments showed loose self-assembly of FP in lipid bilayer highlighting that oligomerization is necessary (but not sufficient) for the fusion activity (Cheng et al., 2003). Recently it has been showed that the membrane fusion requires the concerted action of at least three hemagglutinin trimers (Danieli et al., 1996; Ivanovic et al., 2013; Kielian, 2014).

In recent years, several research groups started to employ atomistic and/or coarse-grained molecular dynamics (MD) simulations to investigate the matter. A variety of atomistic MD studies have been carried out to understand and test the various mechanisms based on the experimental findings. Early atomistic MD studies on the subject highlighted the importance of specific interactions for the anchoring to the membrane and for the adopting of a tilted structure with respect to the normal of the bilayer plane in agreement with experimental findings (Vaccaro et al., 2005). Atomistic simulation using implicit membranes showed that trimer oligomerization of FP determines their reorientation with respect to the membrane that could be of biological relevance (Sammalkorpi and Lazaridis, 2007). In a study of the fusion process in lipid vesicles (Kasson et al., 2010), it has been found that the transition state of this process is defined by the contact of a few lipid tails from the fusing vesicles. This contact occurs when lipid tails protrude into the hydrophilic region, which indicates that this event is a determinant for the fusion process. In the same study, the authors performed simulations of the influenza FP in lipid membranes and found that the peptide promotes lipid tail protrusion in nearby lipids, (Kasson et al., 2010) which has also been observed in other atomistic MD simulation studies (Larsson and Kasson, 2013; Légaré and Lagüe, 2014). These results suggest that the fusion peptide accelerates the fusion process by inducing lipid tail protrusion (Kasson et al., 2010). More recent results suggest the FP not only induces lipid tail protrusion, but also promotes polar head intrusion, i.e., sinking of nearby lipid head groups (Légaré and Lagüe, 2014).

Other atomistic MD simulation studies have also tried to elucidate how the influenza and other viral fusion peptides, such as the fusion peptide from HIV gp41, insert and interact with lipid membranes (Barz et al., 2008; Kasson et al., 2010; Li et al., 2010; Panahi and Feig, 2010; Taylor and Sansom, 2010; Larsson and Kasson, 2013; Baker et al., 2014; Victor et al., 2015). In most of these studies the authors used either an implicit model for the lipid bilayer or a pre-equilibrated membrane in which the peptide was inserted. In most cases the V-shaped structure obtained by Han et al. (2001), using only the first 20 aminoacid residues of HA2, was used and this structure was found to be stable in model membranes.

In a recent study, MD simulations using the closed hairpin structure of the full length influenza FP and an explicit membrane bilayer were performed (Brice and Lazaridis, 2014). In these simulations, the peptide was stable and remained close to the lipid phosphate groups. However, the membrane is a very viscous environment, therefore, the insertion depth, orientation and structure of the peptide are constrained and are not expected to change considerably in the time scale available to current standard atomistic MD simulations.

Due to these limitations, the results obtained in previous MD studies may reflect the initial choices that were made regarding the structure and insertion of the peptide. For these reasons, coarse-grained MD simulations started to be applied for the study of this topic with the aim to extend the temporal scale and consequently enhance the sampling of the relevant events.

Despite the lower resolution, coarse-grained (CG) MD simulations showed agreement with the atomistic ones, confirming, for example, the finding that fusion peptide accelerates the fusion process by inducing lipid tail protrusion (compare Kasson et al., 2010 with Mirjanian et al., 2010) Moreover, improved CG models permitted MD simulation studies to elucidate how the influenza fusion peptides insert and/or interact with lipid membranes (Haria et al., 2014) and to propose that fusion peptides have a role more as stabilizer of pores rather than stalkers along the fusion pathway (Fuhrmans et al., 2009; Fuhrmans and Marrink, 2012; Risselada et al., 2012).

In all of these works, the behavior of a single FP monomer was studied. In this manuscript, we use a simplified model of a tripeptide (TP) monomer of FP (to which we will refer with the acronym TFP; Haria et al., 2014) instead of single FP monomers because each Influenza Hemagglutinin contains three FP molecules in the biological system. Previously, we have discussed the behavior of one TFP monomer in a model lipid membrane, but in this manuscript we report findings targeted at understanding the collective behavior of these TFP monomers on a model lipid membrane. In doing so, we are interested in investigating the fusogenic properties of TFP monomers to understand how they self-assemble in the presence of the membrane and how these self-assembled structures affect and perturb the structure of the phospholipid membrane. Our work can be considered as a contribution to the understanding of initial steps involved in the fusion process as mediated by TFP, with the aim to stimulate discussion and further work.

## 2. Materials and methods

### 2.1. Simulation systems

CG MD simulations were conducted to study the self-assembly of FPs at the interface between water and a POPC bilayer. The sequence of each FP is GLFGAIAGFIENGWEGMIDG. The FP, lipids and water were modeled with the MARTINI CG force field (Marrink et al., 2007; Monticelli et al., 2008). The atomistic representation of the FP monomer was converted to the CG representation following MARTINI procedure (Monticelli et al., 2008). The MARTINI force field for proteins keeps the secondary structure of the protein as rigid, therefore the force field for the FP was modified in order to allow the structure to change conformation. To do so, the force constants for the dihedral angles that make up the backbone of residues 11 GLU, 12 ASN and 13 GLY in the FP were modified in order to allow the peptide to find the two different kink angles observed experimentally (Haria et al., 2014).

**Figure 1:**
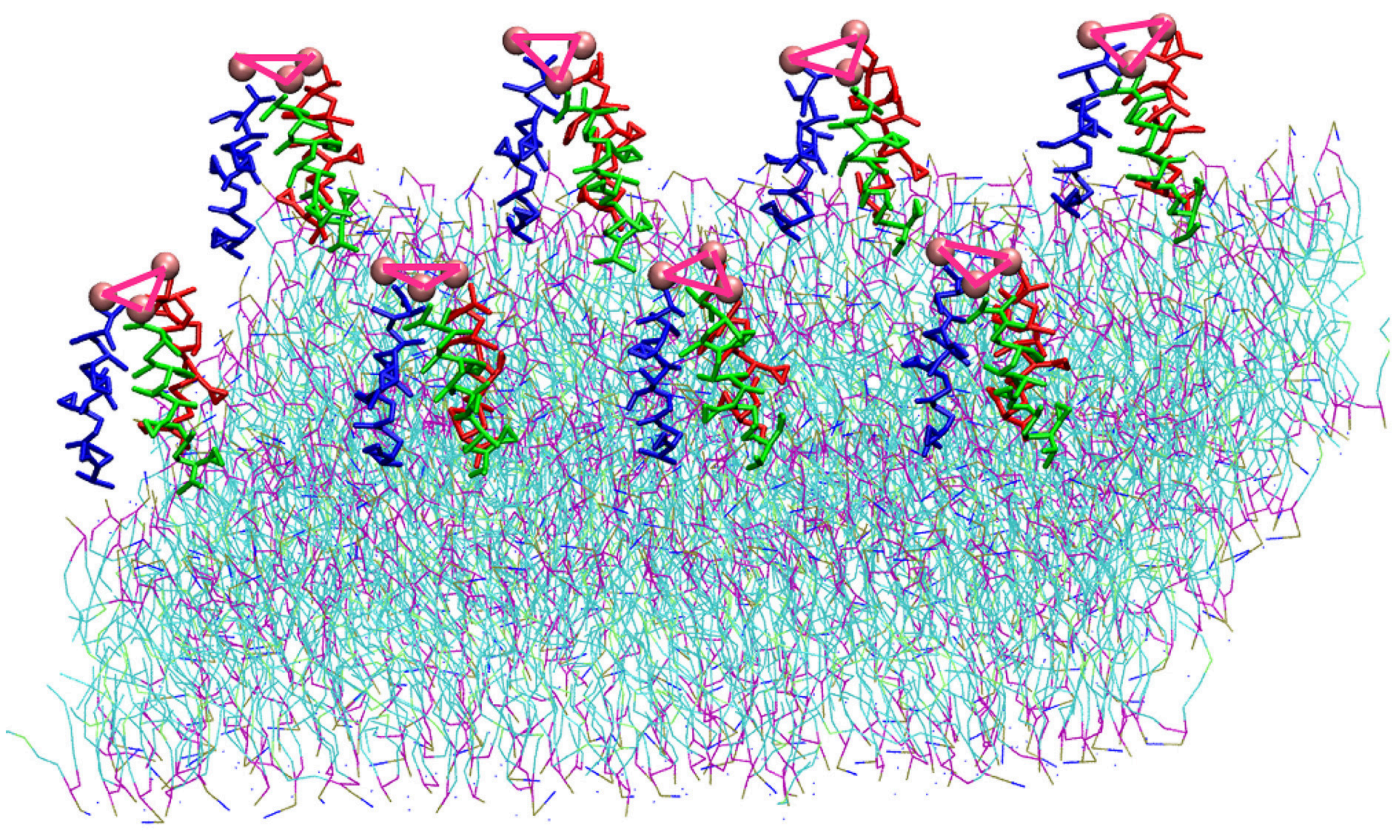
**Representation of the simulation box: 8 TFP monomers interacting with a POPC phospholipid bilayer in an aqueous environment**. Each TFP monomer is composed by three 20-long fusion peptides chain colored in red, blue and green. The beads shown in Van der Waals representation are GLY residues added to bond three peptide chains. Note that the water is not shown so that the details of the TFP monomers and the lipid membrane can be seen.

The TFP monomer was constructed by creating three replicas of the atomistic representation of the single FP monomer and using them to create a coiled-coil complex. The symmetry and orientation of the three helices are analogous to previous work on FP assembly for these peptides (Sammalkorpi and Lazaridis, 2007; Haria et al., 2014). In the HA molecule, this coiled coil is attached to the rest of the molecule and therefore the peptides are not allowed to completely separate and diffuse within the membrane. Therefore, a constraint at the top of the trimer was added to represent this situation. An additional bead representing a GLY residue was added to the C-terminal end of each FP monomer, which was then used to connect the three FP monomers into a TFP monomer by adding harmonic bonds between these additional beads. In order to constrain the monomers into a trimeric structure representative of the atomistic model, the bonds between the additional GLY residues were modeled with a force constant of 5000 kJ mol^−1^ nm^−2^ and an equilibrium length of 1.1 nm and harmonic angle terms were added between the various additional GLY beads such that their equilibrium angle is 60°, and the force constant is 7000 kJ mol^−1^ rad^−2^. These values for the bond and angle force constants are 4 and 10 times greater than the values used for the backbone beads of a peptide, respectively. The pre-assembled TFP monomer was placed in a 4 × 2 grid above a POPC bilayer resulting in a system with 8 FP trimers, 1152 POPC molecule, 17392 water beads for a total of 34,600 beads with a dimension of the box of X = 182.852 Å, Y = 182.852 Å, Z = 112.223 Å (Figure 1).

### 2.2. Simulation methodology

A consistent simulation methodology was used in all simulations, which was similar to that used in Haria et al. (2014). To equilibrate all the systems, a steepest descent energy minimization was first carried out for 1000 steps. Three position restraint simulations were performed after the energy minimization in order to remove any overlapping beads that may still occur as a result of placing the TFP monomers within the membrane. The positions of the TFP monomers were constrained by employing an initially high value of force constant (1000 kJ mol^−1^ nm^−2^) in all directions for the first run. Each consecutive run employed a force constant that was an order of magnitude smaller than the force constant used in the previous simulation. An additional short NVT simulation was performed at 300 K using a Nosé-Hoover thermostat (Nosé, 1984; Hoover, 1985) to keep the temperature constant. The production run utilized the NPT ensemble, with a semi-isotropic Parrinello–Rahman barostat (Parrinello and Rahman., 1981) to keep the pressure constant at 1.0 bar. A leap-frog integrator with a 20 fs time step was used for all simulations. The van der Waals interactions were modeled via a shifted LJ potential with a 1.4 nm cut-off. Similarly, a shifted Coulomb potential with a 1.2 nm cut-off was used to compute electrostatics. Four replicas of the system were ran for 40 μs each, with trajectories written every 0.1 ns. The simulations were performed with GROMACS 4.5.3 (Hess et al., 2008).

### 2.3. Analysis of the simulation

#### 2.3.1. Evolution of the oligomers and their structure

The evolution of the oligomer formation has been monitored identifying all possible TFP dimers within each structure extracted from the replicas. To check the existence of possible TFP dimers we used POPSCOMP (Cavallo et al., 2003; Kleinjung and Fraternali, 2005) which is a software designed to calculate the interaction surface between all components of a given complex structure consisting of proteins, DNA or RNA molecules. We used the functionality of POPSCOMP that permit to perform this analysis on a per-residue level for low-resolution structures (Kleinjung and Fraternali, 2005) as this is the case for our CG MD simulations. One TFP dimer was considered to exist if POPSCOMP identified at least one pair of interacting residues between the two TFP monomers.

For each structure, from the knowledge of the existing TFP dimers, the adjacency matrix for the individual TFP monomers has been built. The adjacency matrices have been analyzed with the Python language software package NetworkX (Hagberg et al., 2008) in order to identify all the possible aggregates as TFP dimers, TFP trimers, TFP tetramers and so on. For each structure the occurrence of each oligomeric state has been calculated simply summing the number of the independent aggregates.

From each replica, the structures of each oligomeric state (TFP dimer, TFP trimer etc.) have been collected with the aim to cluster them and to identify the most populated arrangements. Each oligomeric state has been reconstructed using the minimum convention image. The clustering has been done using the Jarvis–Patrick algorithm (Jarvis and Patrick, 1973) as implemented in GROMACS (Hess et al., 2008).

#### 2.3.2. Orientations/distances of TFP monomers with respect to the membrane

**Figure 2:**
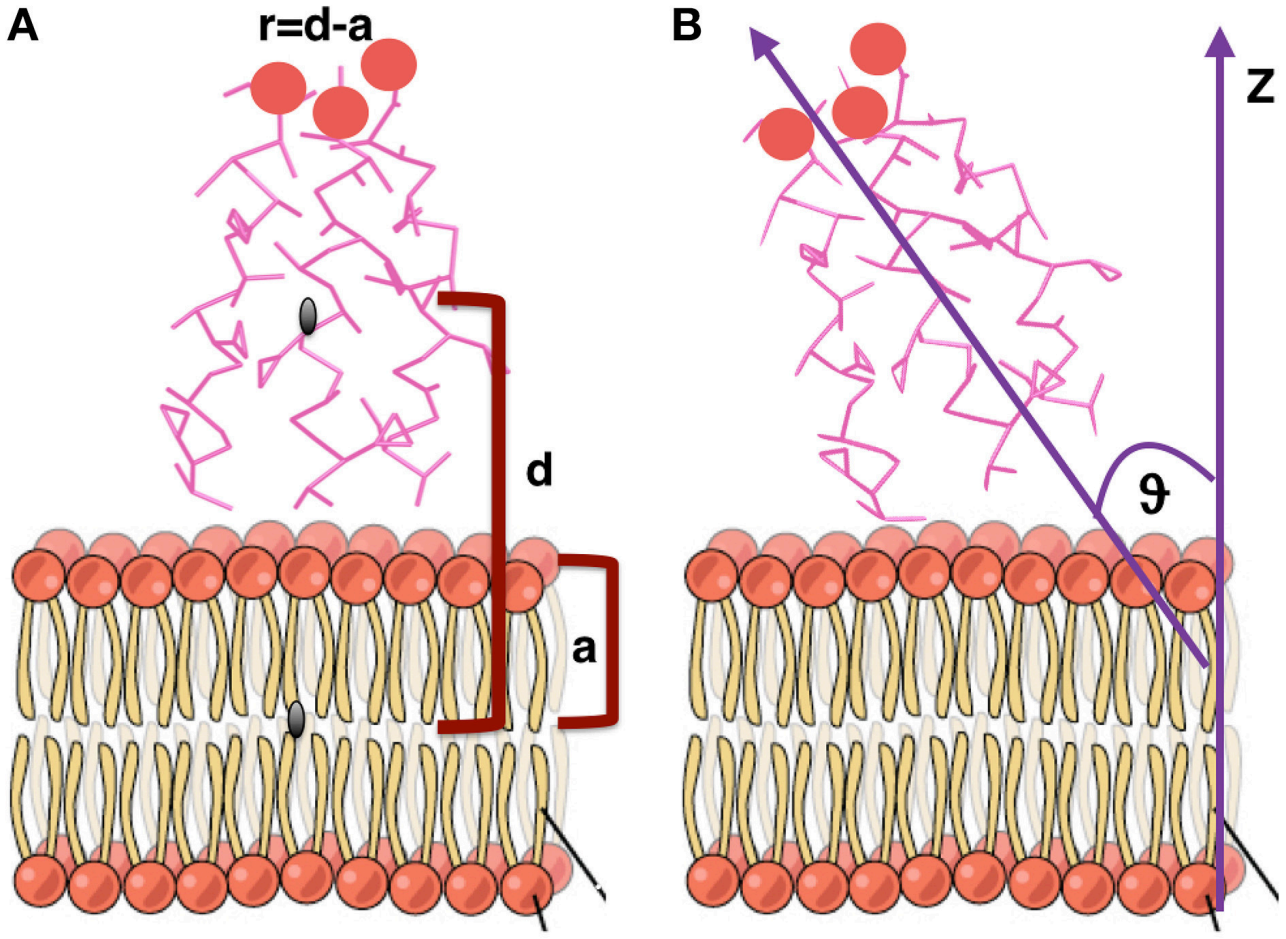
**Graphical representation of the geometrical quantities calculated to estimate orientations and distances of monomers and oligomers with respect to the membrane**. **(A)** Distance from the membrane. **(B)** Orientation with respect to the membrane. Figure adapted from “The Cell Membrane” http://cnx.org/content/m46021/latest/.

In order to calculate the orientation of a TFP monomer with respect to the membrane, the principal inertia axes of a TFP monomer have been calculated using the tools of GROMACS (Hess et al., 2008). The orientation of the TFP monomer with respect to the membrane has been later calculated estimating the angle between the main axis of inertia of the TFP monomer with respect to the z-axis of the membrane (Figure 2B).

The distance of each TFP monomer with respect to the membrane has been calculated with the following formula:

(1) $$\begin{array}{l} r=d-a \end{array}$$

where *d* is the distance, along the z-axis, of the center of mass of each TFP monomer from the center of mass of the membrane and *a* is half of the thickness of the membrane (Figure 2A). This formula has been employed to be able to quantify if the TFP monomers or the oligomeric states are capable of insertion into the membrane during the simulation.

#### 2.3.3. Orientations/distances of oligomers with respect to the membrane

After conducting the analysis of the time evolution of the oligomers, it is possible to identify all the possible TFP dimers, TFP trimers, TFP tetramer, TFP pentamer, and TFP hexamer present in the simulation.

In order to calculate the orientation of each type of oligomer with respect to the membrane, the principal inertia axes of the oligomer have been calculated using the tools of GROMACS (Hess et al., 2008). The orientation of the oligomers with respect to the membrane has been later calculated by determining the angle between the main axis of inertia of the peptides with respect to the z-axis of the membrane.

For each of these oligomers, the distance from the membrane has been calculated using Equation (1) where in this case *d* is the distance, along the *z*-axis, of the center of mass of the oligomer from the center of mass of the membrane.

#### 2.3.4. Curvature, thickness and area per lipid of the membrane

In order to understand the influence of each oligomeric state on the lipid organization, we had to reconstruct the lipid positioning around the oligomer by using the following procedure:

1. for each specific oligomer in each structure the center of mass on the *x*-*y* plane has been calculated;
2. each center of mass on *x*-*y* plane of the oligomer (in each structure) is used as the “center” to apply the minimum image convention for the reconstruction of the phospholipid bilayers as placed just below the oligomer;
3. each phospholipid bilayer and the corresponding oligomer are translated to have their center of mass on the *x*-*y* plane in 0, 0.

Following this procedure, for each possible oligomeric state, a trajectory of the corresponding phospholipid bilayers is created. The resulting trajectories contain information of the properties of the system when an oligomer of a given size is present, but are not necessarily consecutive states of a given trajectory as time progresses. Therefore, we are not able to determine time-dependent properties of the lipid bilayers from this analysis, but we are able to calculate properties of the phospholipid bilayer simply averaging the results obtained in each structure. Each of these trajectories has been analyzed to calculate curvature, thickness and area per lipid of the phospholipid bilayer as influenced by the aggregates.

The analysis has been conducted using the tool *g_lomepro* (Gapsys et al., 2013). Briefly, *g_lomepro* maps the lipid beads onto a grid. The calculation of the membrane curvature is based on the assignment of the grid points to the corresponding lipid coordinates along the normal to the bilayer, obtaining in this way the coordinates over a surface S = S (x,y). From the knowledge of the surface, the first and the second order derivatives as well as the normals to the surface at every grid point are calculated to obtain the coefficients of the first and second fundamental form and consequently the mean curvature (Gapsys et al., 2013).

The results of this analysis have been post-processed to produce the heat map figures for the curvatures and the thickness induced by each aggregate state.

## 3. Results

### 3.1. Peptides oligomerization

We have performed CG MD simulations of four replicas of the system. For each replica we have studied the oligomerization process of HA TFP monomers at the interface between water and the phospholipid bilayer. We have performed 40 μs of MD simulation for each replica for a total of 160 μs. For all replicas, we have analyzed the oligomerization process during the simulations and all replicas show a common behavior on average.

The maximum sized oligomer formed is the TFP hexamer, but the TFP pentamer has a longer life time as compared to the TFP hexamer, meaning that the TFP pentamer is the largest stable oligomer. However, from a statistical point of view, a common feature in all of the replicas is that the TFP trimer is the most stable oligomer formed. Another common feature is that the formation of the oligomers is based on the formation of TFP trimers and once this core is formed then additional TFP monomers will bind to it until a stable larger oligomer is formed. The oligomers are dynamic entities with a fluctuating size during the simulation, because TFP monomers bind to the other TFP monomers. However, once a TFP trimer is formed, we do not observe a TFP monomer unbinding from a TFP trimer resulting in it becoming only a TFP dimer or completely breaking up into three TFP monomers, while we do observe TFP monomers binding to TFP trimers to create a larger oligomer. This means that our simulations show that TFP trimers and tetramers are the most stable aggregate states at the interface with the membrane. Little is known in the literature (at the best of our knowledge) about the residues mediating the formation of TFP trimers. From the calculation of the average number of internal contacts in the TFP trimers, we found that the most important residues involved in the stabilization of the oligomers are ALA 5, ILE 6, PHE 9, ILE 10, GLY 13, TRP 14, MET 17 and ILE 18 (see Figure 5). This list of residues nicely fit with the phase periodicity of the helical structure of hemagglutinin fusion peptides. This result is relevant because it shows the importance of the peptide helical structure for the mediation of the fusion process.

**Figure 3:**
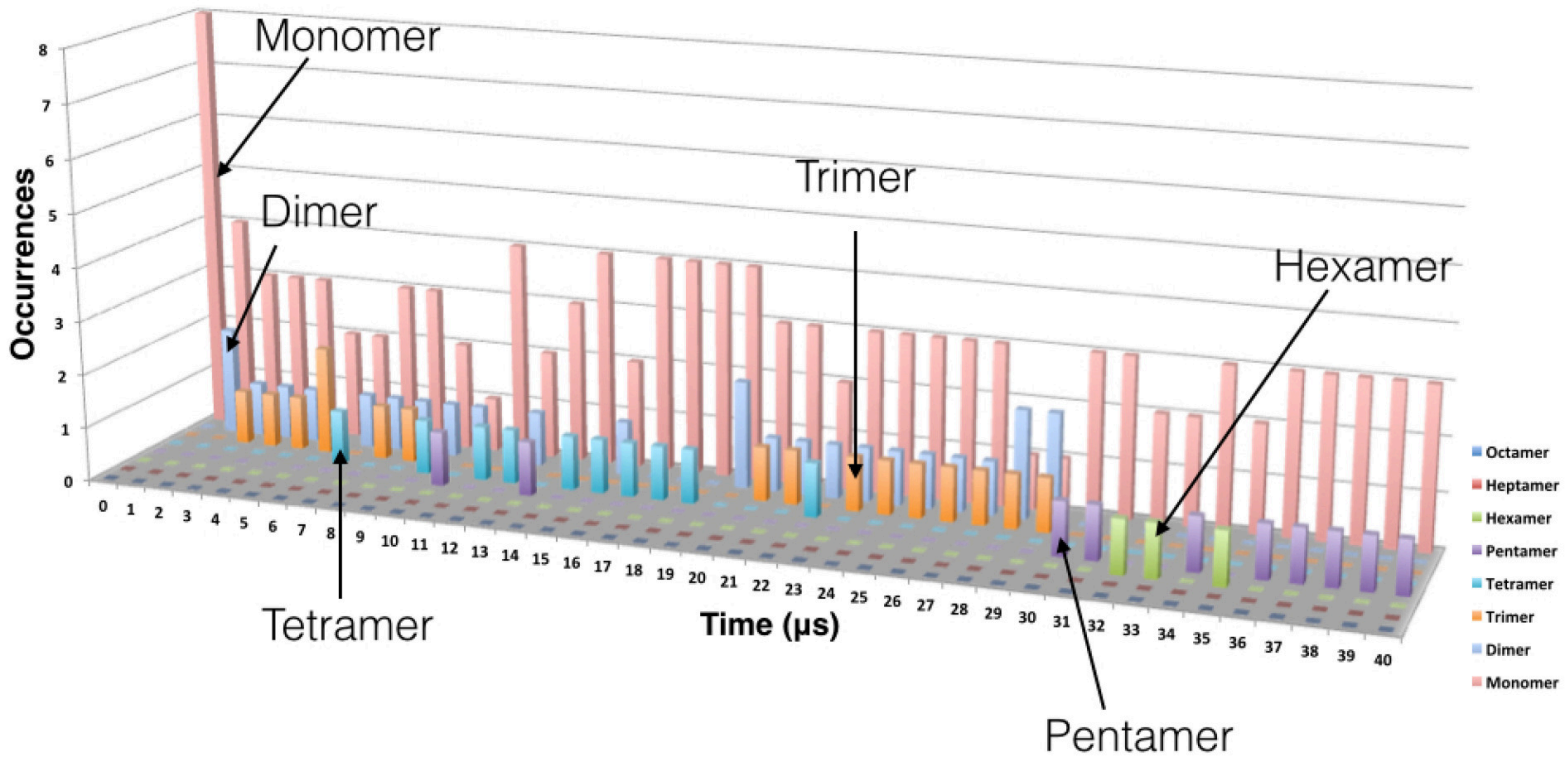
**Evolution of the oligomer formation as a function of time**. Oligomerization was measured every microsecond. The data shown here is from one replica but is representative of the behavior observed in the other replicas. The monomers are represented in pink, the dimers in cyan, the trimers in orange, the tetramer in blue, the pentamer in violet and the hexamer in green.

The process of the oligomer formation is very dynamic and it is showed in Figure 3 for one representative replica. Figure 3 shows the number of each type of oligomer for every microsecond of the CG MD simulation. In the initial configuration, there are eight TFP monomers in the box. From the second μs there is the formation of TFP dimers and in the third μs a TFP trimer appears. From the third μs to the 7th μs, the biggest oligomer is a TFP trimer, that is in fact the core of the oligomerization process observed in our simulations.

**Figure 4:**
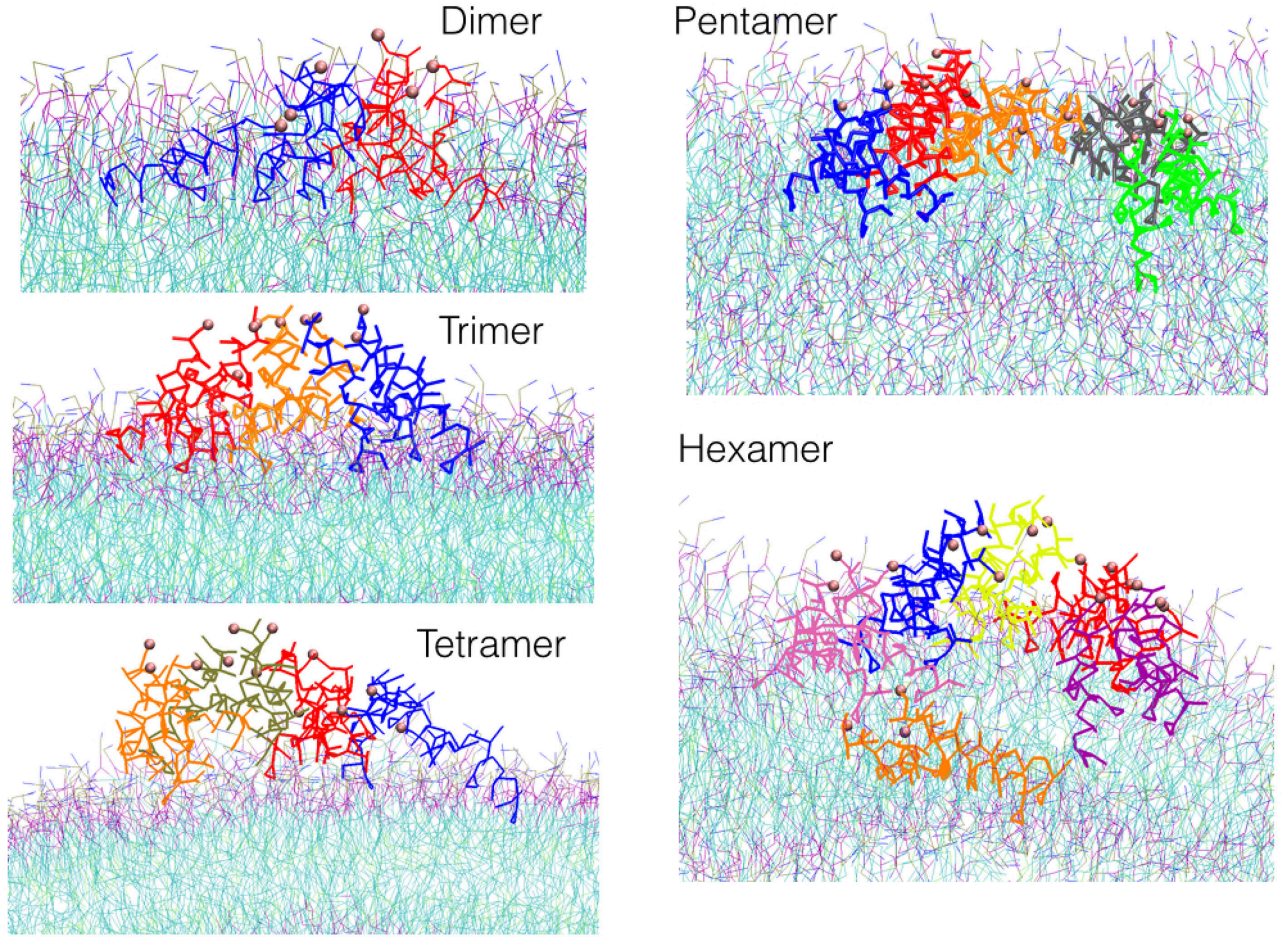
**Snapshots of the most representative structure for each oligomer found during the simulations**.

From the 7th μs, a TFP monomer becomes attached to the trimer leading to the formation of a TFP tetramer. At the 11th μs, a TFP monomer attached to the TFP tetramer leading to the formation of the TFP pentamer. From the 15th μs until the 30th μs, various oligomeric states including a TFP pentamer, TFP trimers, TFP dimers and TFP monomers are present within the simulated system. From the 33rd μs, a TFP monomer binds to a TFP pentamer to form a TFP hexamer, and two TFP monomers remain unbound. The TFP hexamer remains stable for two μs and than a TFP monomer unbinds from the TFP hexamer resulting in a stable TFP pentamer and three TFP monomers. Figure 4 shows the most representative arrangement of oligomers as obtained by the use of the clustering algorithm.

**Figure 5:**
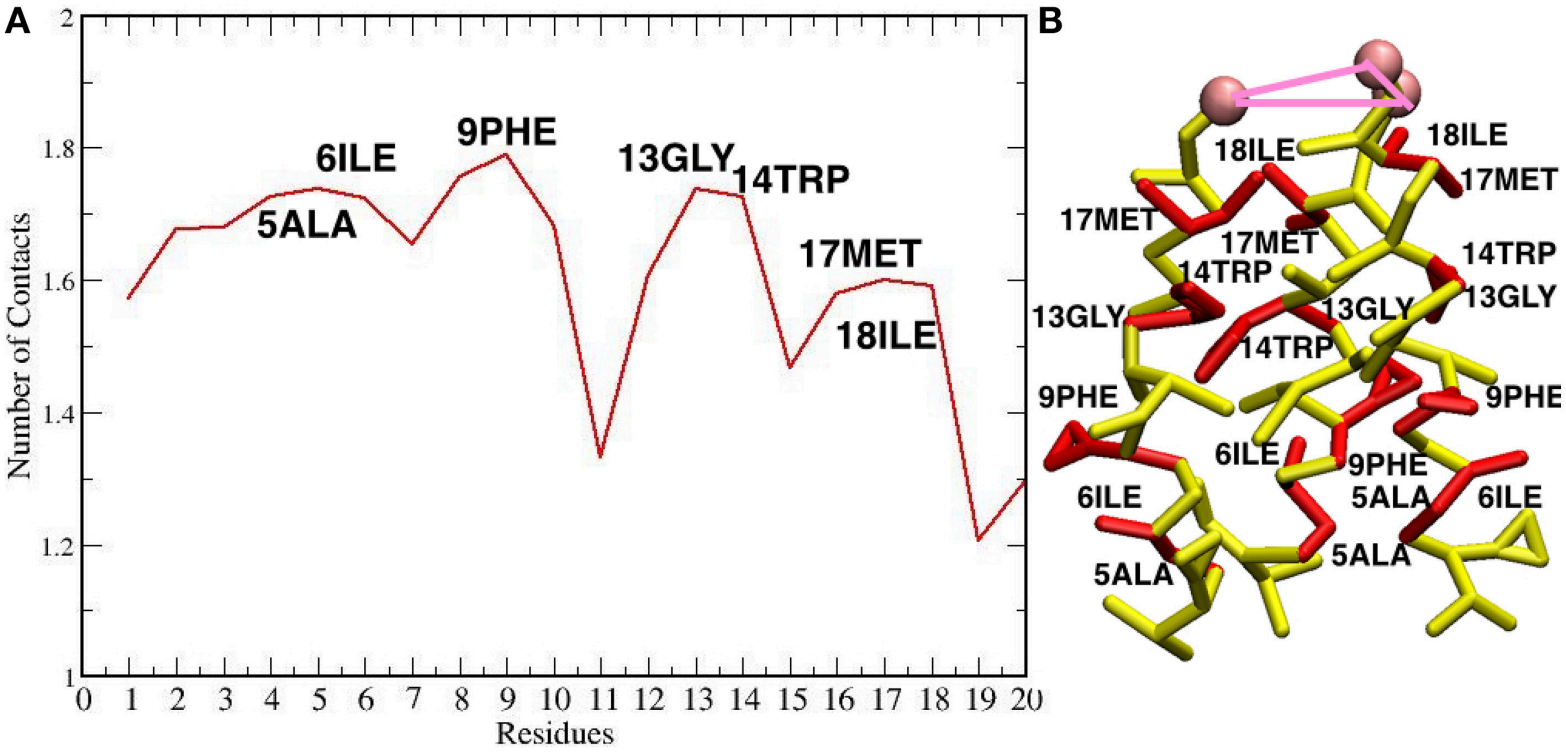
**(A)** Plot of the number of contacts of the residues of each TFP monomer during the self-assembly of TFP trimers. **(B)** The residues that are interacting the most during the self-assembly of TFP trimers are highlighted in red on the TFP monomer structure.

The formation of the TFP trimer as the core and nucleation center for the growth of the oligomer has been observed in all simulation replicas. See the Supplementary Material for the evolution of the oligomer formation as observed within the other replicas.

### 3.2. Penetration of the TFP monomers and oligomers in the membrane

**Figure 6:**
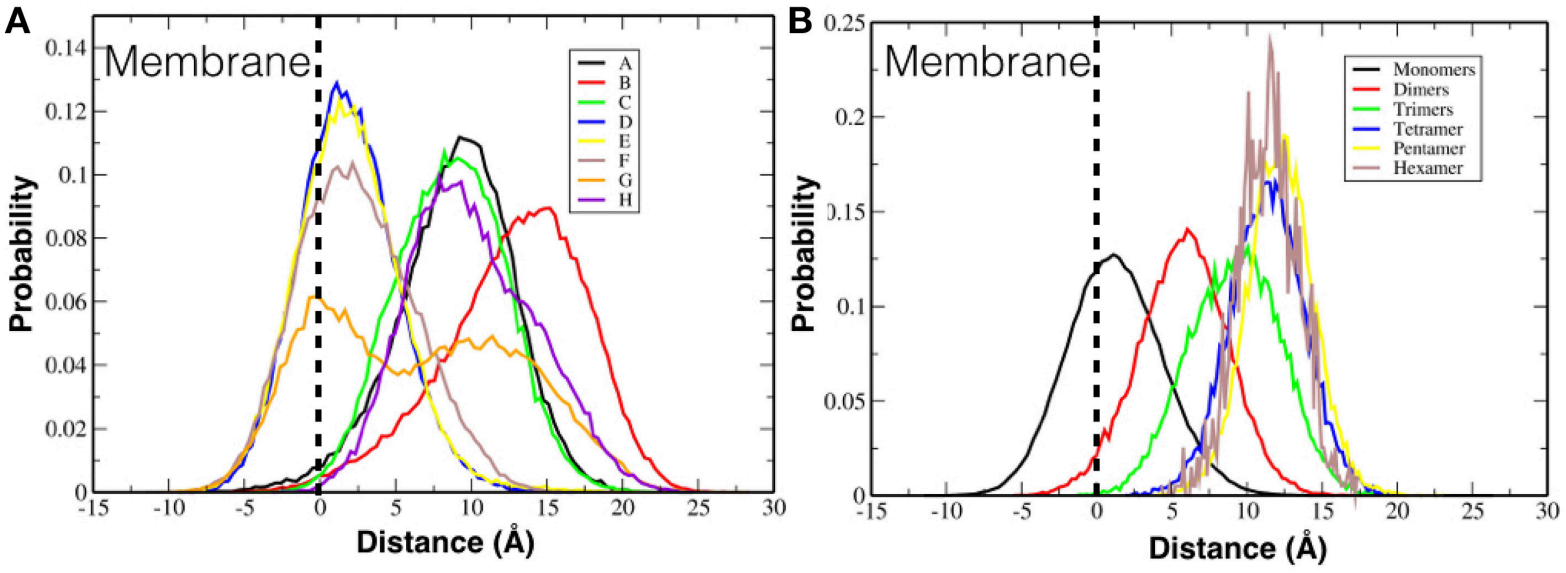
**Probability distribution of the positioning of FPs with respect to the membrane surface**. **(A)** Probability distribution is calculated for each single TFP monomer. **(B)** Probability distribution is calculated for the oligomeric state.

We investigated if the FP peptides were able to penetrate into the membrane. Figure 6A shows the histograms of the distance of the center of mass of every single TFP monomer, without taking into account if they are part of a oligomer or not, with the center of mass of the membrane. Several TFP monomers were able to penetrate about 8 Å into the lipid membrane (−8 Å in the plot, where 0 Å denotes the lipid membrane interface and values greater than 0 Å represent the peptide being outside of the membrane) but most of the time the TFP monomers were found lying on the surface of the membrane. In fact the peaks of the histograms are at 2 Å, 10 Å, and 15 Å. We also calculate the distance between the center of mass of every oligomer type and the center of mass of the membrane. This is showed in Figure 6B. If we consider the single oligomers as opposed to the individual TFP monomers we can identify four peaks: at 2 Å for the TFP monomers, at 6 Å for the TFP dimers, at 10 Å for the TFP trimers and finally at 12 Å for the TFP tetramer, the TFP pentamer and the TFP hexamer. It is possible to say that peptides that have distances less than zero in Figure 6A are due to transient arrangements of single TFP monomers. In fact, even if TFP monomers (as a single entity) penetrated more deeply into the membrane with respect to the other oligomers, this state is not stable, because the peaks of the histogram is never inside the membrane but is ~2 Å from the membrane's surface. The configurations where the TFP monomers and TFP dimers penetrated several Angstroms into the membrane belongs to configurations in the tail of the probability distribution of the TFP monomers positioning, so they are not stable configurations. This means that also for the TFP monomers the most probable configurations are on the surface of the membrane.

These geometrical arrangements are characterized by well-defined protein-lipid interactions. Analysing the average number of contacts that each residue of TFP trimers form with the membrane, we found that the residues GLY 1, LEU 2, GLY 8 and PHE 9 are the most important, whereas the least important are GLU 11 and GLU 15 (see Figure 10). This finding is in agreement with the known residues important for the membrane fusion process and fit with the phase periodicity of the helical structure of hemagglutinin fusion peptides (Skehel and Wiley, 2000). The same is observed for the other TFP oligomers. As in the case of the peptide oligomerization results, this finding is relevant because it shows the importance of the peptide helical structure for the mediation of the fusion process.

In conclusion, during the formation of the oligomers, neither individual TFP monomers nor oligomers penetrate the membrane, because their amphipathic nature causes them to prefer to remain at the membrane's interface. The residues responsible for the interaction with the membrane are the same residues known to play a fundamental role in the membrane fusion process.

### 3.3. Orientation of the TFP monomers and oligomers with respect to the membrane

We investigated if the peptides were able to assume specific orientations with respect to the membrane.

**Figure 7:**
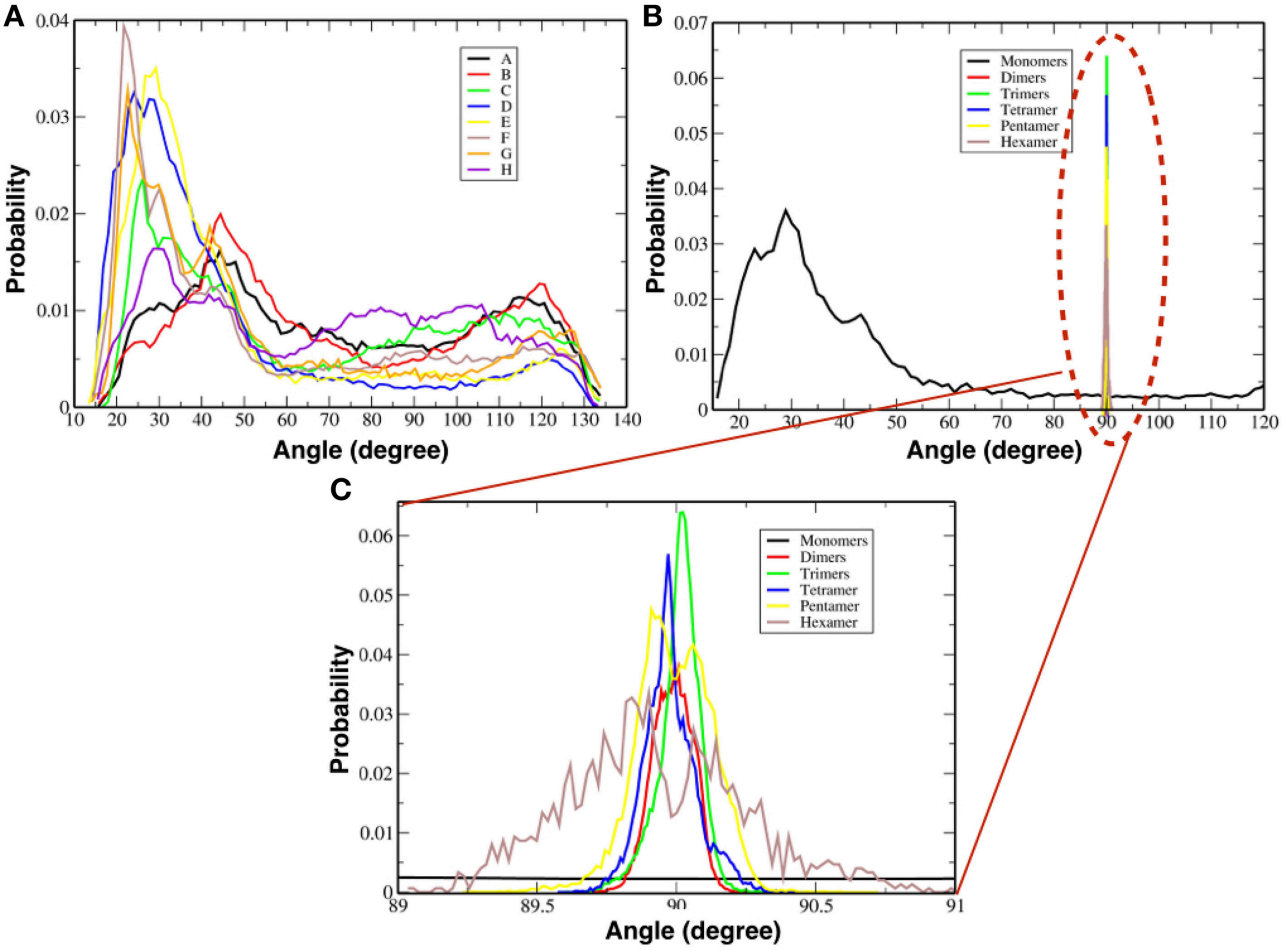
**Probability distributions of the orientation of monomers and oligomers**. **(A)** Probability distributions for each single TFP monomer. **(B)** Probability distributions for the oligomeric state. **(C)** Close-up view of the probability distributions as calculated for oligomeric states observed in our simulations.

Figure 7A shows the probability distribution for the orientations of each TFP monomer with respect to the membrane without taking into account if they are participating in the formation of an oligomer. From the plot, it is possible to see that there is a peak at 30° and a long tail that extends until 130°. This means that any orientation defined by angles within that range is possible with a preferred orientation at 30°.

Figure 7B shows instead the probability distribution orientations of the various oligomeric states considering also the TFP monomers when they are not forming any oligomer. As shown for the single monomers when they are not forming any oligomer, any orientation is possible with a peak at ~30°. Whereas for the TFP oligomers the relative orientation assumes only specific values centered around ~90°. In order to clarify this discrepancy, the probability distributions of the TFP oligomers have been shown as well in Figure 7C, where it is clearly shown that the most probable orientations is ~90°. Therefore, the TFP oligomers are laying parallel to the membrane's interface during the majority of the simulation.

**Figure 8:**
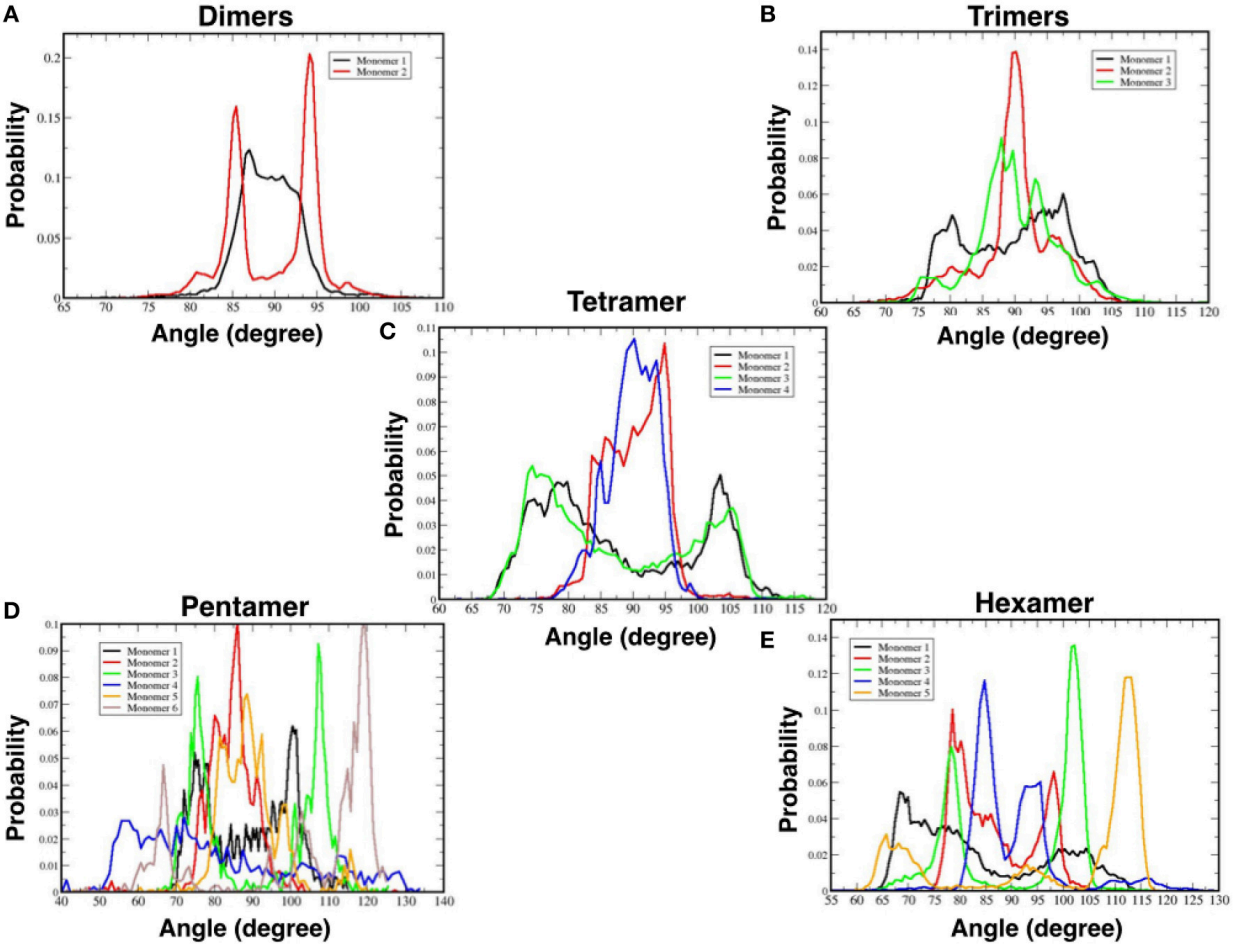
**Probability distributions of the orientations of TFP monomers while a part of a specific oligomer: (A)** TFP dimers, **(B)** TFP trimers, **(C)** TFP tetramers, **(D)** TFP pentamers, and **(E)** TFP hexamers.

We also measured the orientation of the TFP monomers that make up specific oligomeric states and the results are presented in Table 1 and Figure 8. As the oligomer size increases, the spectra of the possible orientations of the TFP monomers composing it, is as broader as possible from the TFP dimers to the TFP pentamer. In the case of the TFP hexamer, the spectra is narrower than the one of the TFP pentamer: we justify this by the short life time observed for the TFP hexamer, that does not permit the sampling of all the possible orientations of its constituent monomers.

**Table 1:** **Preferred orientations in degrees (°) of the TFP monomers while being a member of a specific oligomeric state**.

	**TFP 1**	**TFP 2**	**TFP 3**	**TFP 4**	**TFP 5**	**TFP 6**
TFP Dimer	85/95	87				
TFP Trimer	80/98	90	90			
TFP Tetramer	75/105	75/105	95	90		
TFP Pentamer	67/103	80/98	78/102	85/95	65/113	
TFP Hexamer	75/102	85	75/108	55	90	68/103/120

*Distributions of the orientations for each oligomeric state are shown in Figure 8*.

Similar results have been obtained for the other replicas (see Supplementary Material).

### 3.4. Fusion peptides induce curvature on the membrane and decrease its thickness

**Figure 9:**
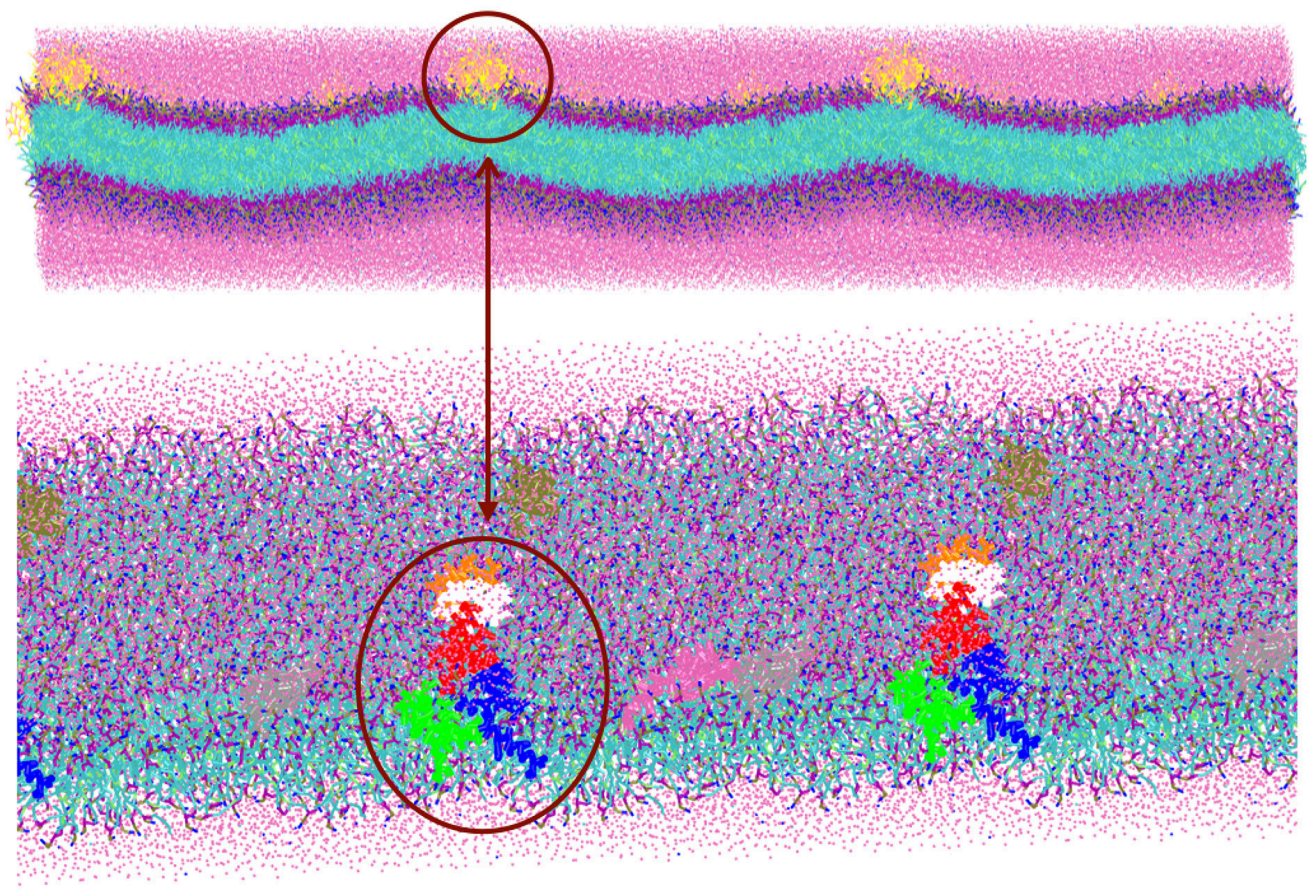
**View of the membrane curvature induced by the presence of a TFP pentamer**.

While the peptides are assembling to form the different oligomers they are inducing curvature on the membrane. Figure 9 shows the heat maps of the curvature induced to the membrane by each oligomeric state for one representative replica. It is possible to observe that, while the oligomers become larger in size, this is paralleled by a larger curvature of the membrane in correspondence of the oligomer center of mass. The curvature of the membrane increased with the size of oligomer (Figure 9). The projections of the positions of the oligomers during the simulations onto the curvature heat map are shown in Figure 11 (dotted black points). For TFP dimers, TFP trimers, and TFP tetramers the maximum of the membrane curvature is localized in correspondence to the oligomers positions. With the formation of TFP pentamers and TFP hexamers the membrane curvature becomes more spread and not directly correlated with the position of the oligomers. As a consequence of this, it appears that TFP pentamers and TFP hexamers generated a perturbation which results in a “ripple” effect within the membrane. Analysis of the thickness of the membrane shows (Figure 12) that while the oligomers become larger the membrane thickness decreases accordingly in correspondence to the oligomers positions.

**Figure 10:**
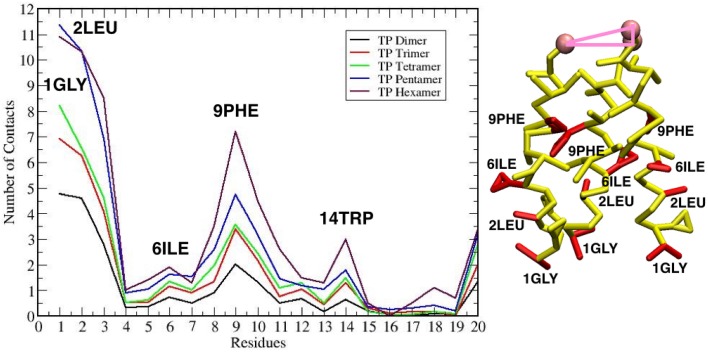
**Left:** Plot of the number of contacts of the residues of each TFP monomer with the membrane. **Right:** The residues that are interacting the most with the membrane are highlighted in red on the TFP monomer structure.

**Figure 11:**
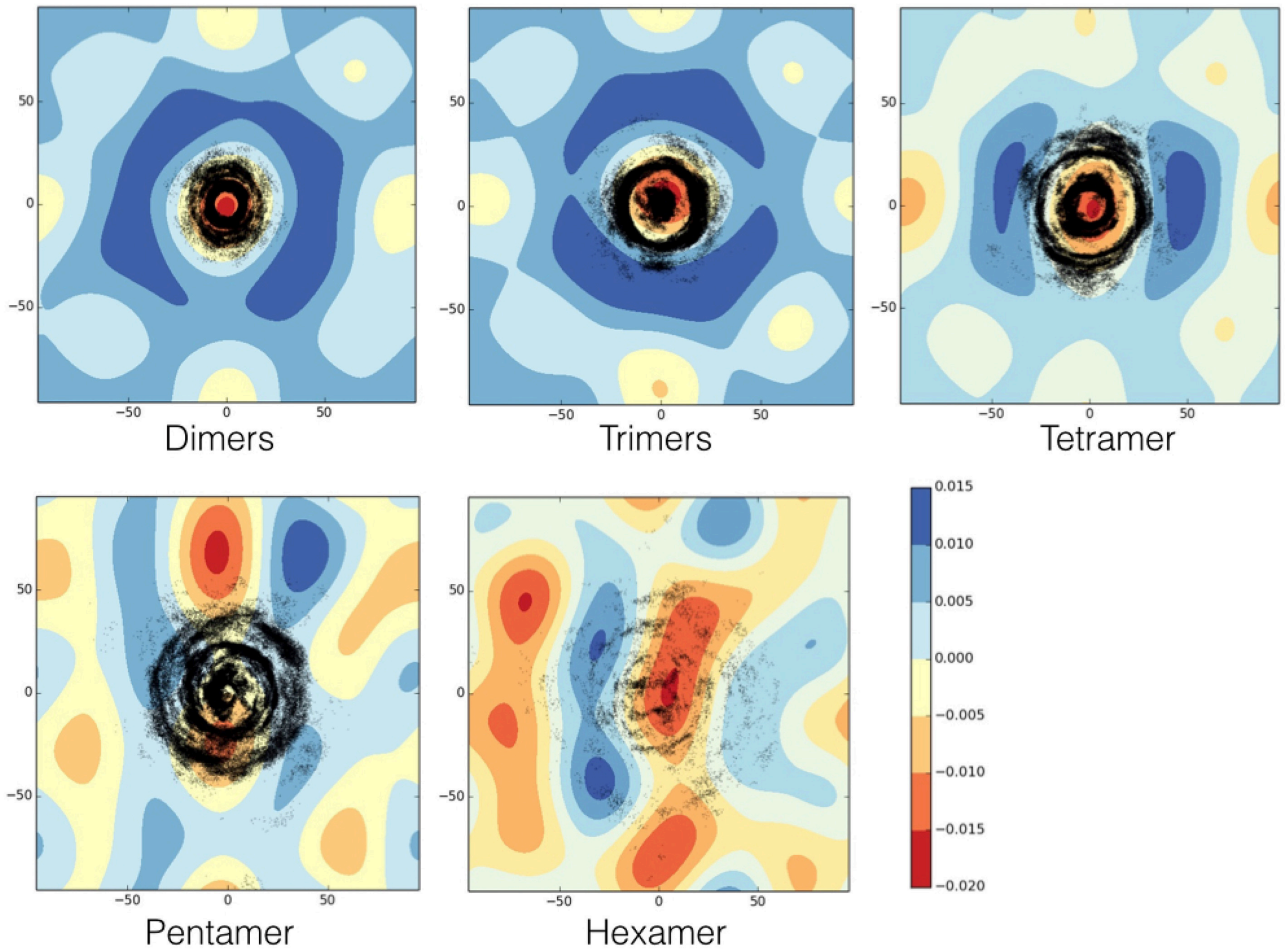
**Heat map of the membrane curvature as induced by each oligomeric state**. Black dots correspond to the position of the center of mass of each monomer belonging to the oligomer. The units for membrane curvature are [nm^−1^].

**Figure 12:**
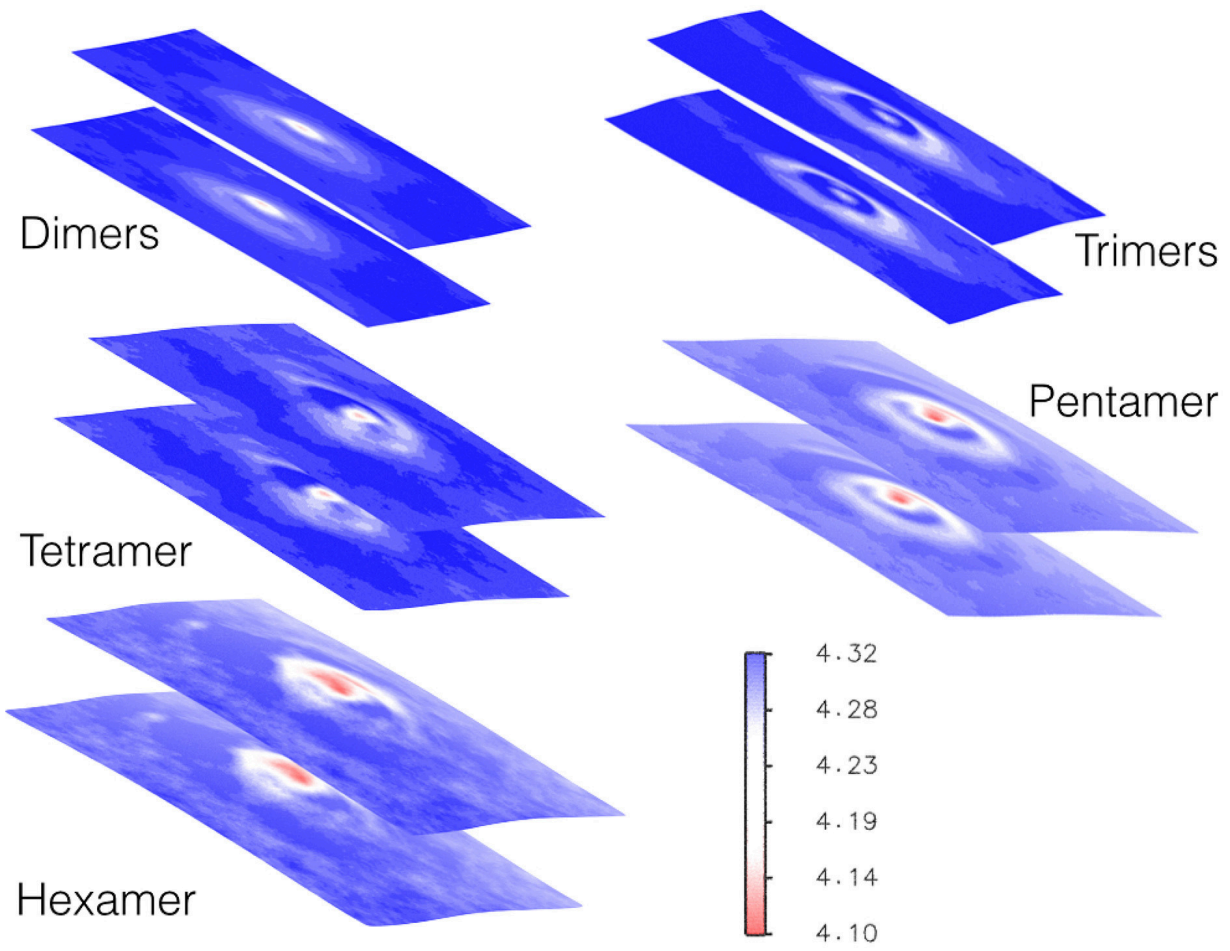
**Heat map of membrane thickness as induced by each oligomeric state**. The units for the membrane thickness are [nm].

## 4. Discussion

We used for the first time a model of a TFP monomer as an approximation of the HA complex in order to take into account that each Influenza Hemagglutinin contains three FPs and we focused our attention on their fusogenic properties. By using this model we were interested in investigating the collective behavior of these TFP monomers on a model lipid membrane and their mechanism of assembly within the membrane by CG MD simulations to understand the possible implications for the membrane fusion process. Our long CG MD simulations showed that the 8 TFP monomers in our system self-assembled to form different oligomers. The largest stable oligomeric assembly was the pentameric state of TFP monomers.

Our simulations showed that the oligomer formation process was based on a formation of a core, a TFP trimer. Once the core was formed, it remained stable and all the other larger oligomers were formed by TFP monomers attaching to the core. The oligomers grew in size in this manner until hexameric oligomers were formed, but we never observed the formation of oligomers containing 7 or 8 TFP. Our simulations showed that, during the oligomer formation process, several oligomers of TFP trimers formed during the assembly were not capable of insertion into the phospholipid bilayer, but only capable of transient penetration, so TFP trimers formed the variety of oligomeric states at the interface between water and the phospholipid bilayer in agreement with previous atomistic and coarse-grain MD studies (Vaccaro et al., 2005; Brice and Lazaridis, 2014; Haria et al., 2014). These findings highlight the amphipathic nature of FP and are in agreement with the generally accepted mechanism of action proposed for them that does not imply a deep insertion into the membrane, but rather a perturbation of its ordered state. Our results seem to show that the interplay of primary, secondary and tertiary structures of the fusion peptides leads to the formation of TFP trimers to facilitate the membrane fusion process. In fact all of the information contained in the hierarchic structural organization of the fusion peptides leads to the formation of strong interactions between the FP and the membrane and tight interactions inside TFP trimers which may be responsible for the anchoring of the TFP trimers into the membrane. The formation of self-assembled structures is in line with steady state fluorescence experiments that showed evidence of loose self-assembly of FP in lipid bilayer, that is necessary (but not sufficient) for their fusion activity (Cheng et al., 2003).

We investigated the geometric organization of the single TFP monomers and of the different oligomers. The single TFP monomers, when not participating to a specific oligomerization state, assumed random orientations with the membrane whereas the different oligomers of TFP monomers assumed the predominant orientation of 90°, such that they lay on the membrane surface with their main axis parallel to it. The probability distributions of the single TFP monomers orientations, if not participating to the formation of an oligomer, have a small maximum at 30°, in agreement with early atomistic MD studies (Vaccaro et al., 2005). The TFP monomers participating to a specific oligomer presented a spectrum of orientation that is broader and broader as the oligomer increases.

The second aim of our work was to investigate how these self-assembled structures affect the structure of the lipid membrane. Our work represents a further step with respect to previous computational investigations because we made use of a model of TFP monomers, which should be more realistic than use a single FP to describe the self-assembly process and membrane distortion induction (Fuhrmans et al., 2009; Fuhrmans and Marrink, 2012; Risselada et al., 2012; Haria et al., 2014). We did not make use of specific geometric arrangements of FP that are known to lead to the formation of specific phases in the phospholipid bilayer and fusion pores as was previously done by Marrink's group (Fuhrmans et al., 2009; Fuhrmans and Marrink, 2012; Risselada et al., 2012). We found that each oligomeric state is capable of inducing specific distortions to the membrane that were more pronounced as the oligomer size increased. These distortions were evident from the analysis of the curvature and thickness of the membrane. Each oligomer induced curvature within the membrane, and the curvature increased with the size of the oligomer. For the TFP pentamers and the TFP hexamers, the curvature was larger and also spread over the membrane surface, not directly localized in correspondence to the oligomer center of mass. It appears that the TFP pentamers and the TFP hexamers induced a long ranged perturbation that affected the entire membrane. The results obtained for the curvature correlate with the outcomes of the membrane thickness. In fact, the effect of the oligomer on the thickness of the membrane increases with the size of the oligomer, inducing a thinning in the membrane where the minimum thickness of the membrane is generally found at the oligomer's center of mass. The anchoring to the membrane by TFP is accomplished thanks to the double action of (i) the interaction of some residues with membrane known to be fundamental for the fusion process (namely Gly-1, Leu-2, and Gly-8) and (ii) the internal stabilizing interactions of TFP. The anchoring to the membrane by TFP trimers or tetramers leads to a remarkable curvature of the membrane. As it is possible to see from Figures 9, 11, TFP oligomers induce globally a positive curvature in agreement with recent CG MD findings (Fuhrmans et al., 2009; Fuhrmans and Marrink, 2012; Risselada et al., 2012), whereas, in correspondence of the oligomers' center of mass, the curvature is negative as observed recently for FP in micelles with NMR experiments (Smrt et al., 2015).

## 5. Conclusion

The presented computational work showed the highly dynamic nature of FP oligomeric formation and their capability to distort a model lipid membrane as a result of their propensity to self-assemble. The oligomeric states are characterized by well-defined properties of their internal geometric organization and of their arrangement with respect to the phospholipid membrane. This well-defined geometrical organization is shown to influence the structure of the phospholipid bilayer by inducing curvature and thinning within the membrane. The well-defined geometrical organization is due to key interactions with the membrane and within TFP oligomers in agreement with experimental data (Skehel and Wiley, 2000; Han et al., 2001; Lai and Tamm, 2007). The propensity to self-assemble has previously been observed by steady state fluorescence experimental data (Cheng et al., 2003).

The outcomes of the present study could provide a working hypothesis of the mechanism of FP action and could serve as starting point for further computational studies of the subject. The presented work seems to suggest that the interplay of primary, secondary and tertiary structures of fusogenic peptides leads to the formation of oligomeric states composed of at least a TFP trimer, that could induce relevant deformations in the host membrane and determining an anchoring to it. Moreover once TFP pentamers and TFP hexamers were formed these were able to create a perturbation to the whole membrane and a decreasing of the thickness which occurs in parallel to a consequent halt in oligomer growth. In our simulations the growth stopped when the size of the oligomer was large enough to induce distortion of the membrane.

As a general outcome this work showed the potential of CG MD simulations as a tool to probe the mechanisms of oligomerization of self-assembling biomolecules.

## Author contributions

Conceived and designed the experiments: CL and FF. Performed the experiments: FC. Analyzed the data: FC and ES. Contributed reagents/materials/analysis tools: FC and ES. Wrote the paper: FC, ES, CL, and FF.

## Funding

FC research was founded by Swiss National Science Foundation (SNSF) project P2BEP2_148877. FF research was supported by the Biotechnology and Biological Sciences Research Council (BB/H018409/1).

### Conflict of interest statement

The handling editor Piero Andrea Temussi declares that, despite being a visiting scientist at the same institute as the authors Franca Fraternali, Francesca Collu, and Christian Douglas Lorenz. The authors declare that the research was conducted in the absence of any commercial or financial relationships that could be construed as a potential conflict of interest.

## Supplementary material

Supplementary Data includes figures describing for each replica: evolutions of the oligomer formation during the time for every microsecond of simulation; probability distributions of positioning of peptides with respect to the membrane surface; probability distributions of monomers and oligomers orientations with respect to the membrane surface; heat maps of the membrane curvature as induced by each type of oligomeric state; heat maps of the membrane curvature as induced by each type of oligomeric state with projections of the positioning of each monomer participating to the oligomeric state.
