# Supplementary material for: Assembly of Influenza Hemagglutinin Fusion Peptides in a Phospholipid Bilayer by Coarse-grained Computer Simulations
Source: Front Mol Biosci. 2015 Nov 18;2:66. doi: 10.3389/fmolb.2015.00066 (PMC4649048; doi:10.3389/fmolb.2015.00066)
Supplement: Supplementary file 1 [file DataSheet1.PDF]

Supplementary Material: Assembly of Influenza hemagglutinin fusion peptide trimers in a phospholipid bilayer

Collu Francesca [1], Spiga Enrico [2], Lorenz Christian D. [3,*] and Fraternali Franca [1,*]

[1] *Randall Division of Cell and Molecular Biophysics, King's College London, London, UK SE1 1UL*
[2] *The Francis Crick Institute, Mill Hill Laboratory, London, NW7 1AA UK*
[3] *Materials Research Group, Department of Mechanical Engineering, King's College London, London, UK WC2R 2LS*

Correspondence*:
Fraternali Franca
Randall Division of Cell and Molecular Biophysics, King's College London, London, UK SE1 1UL , franca.fraternali@kcl.ac.uk

Lorenz Christian D.
Materials Research Group, Department of Mechanical Engineering, King's College London, London, UK WC2R 2LS, chris.lorenz@kcl.ac.uk

1 SUPPLEMENTARY FIGURES

We have performed CG MD simulations of four replicas of the system. In what follows for each replica, beside the one presented in the main text, the reader can find:

- Evolution of the oligomer formation during the time for every microsecond of simulation
- Probability distribution of positioning of peptides with respect to the membrane surface
- Probability distributions of monomers and oligomers orientations with respect to the membrane surface
- Heat map of the membrane curvature as induced by each type of oligomeric state
- Heat map of the membrane curvature as induced by each type of oligomeric state with projections of the positioning of each monomer participating to the oligomeric state

1.1 1ST REPLICA

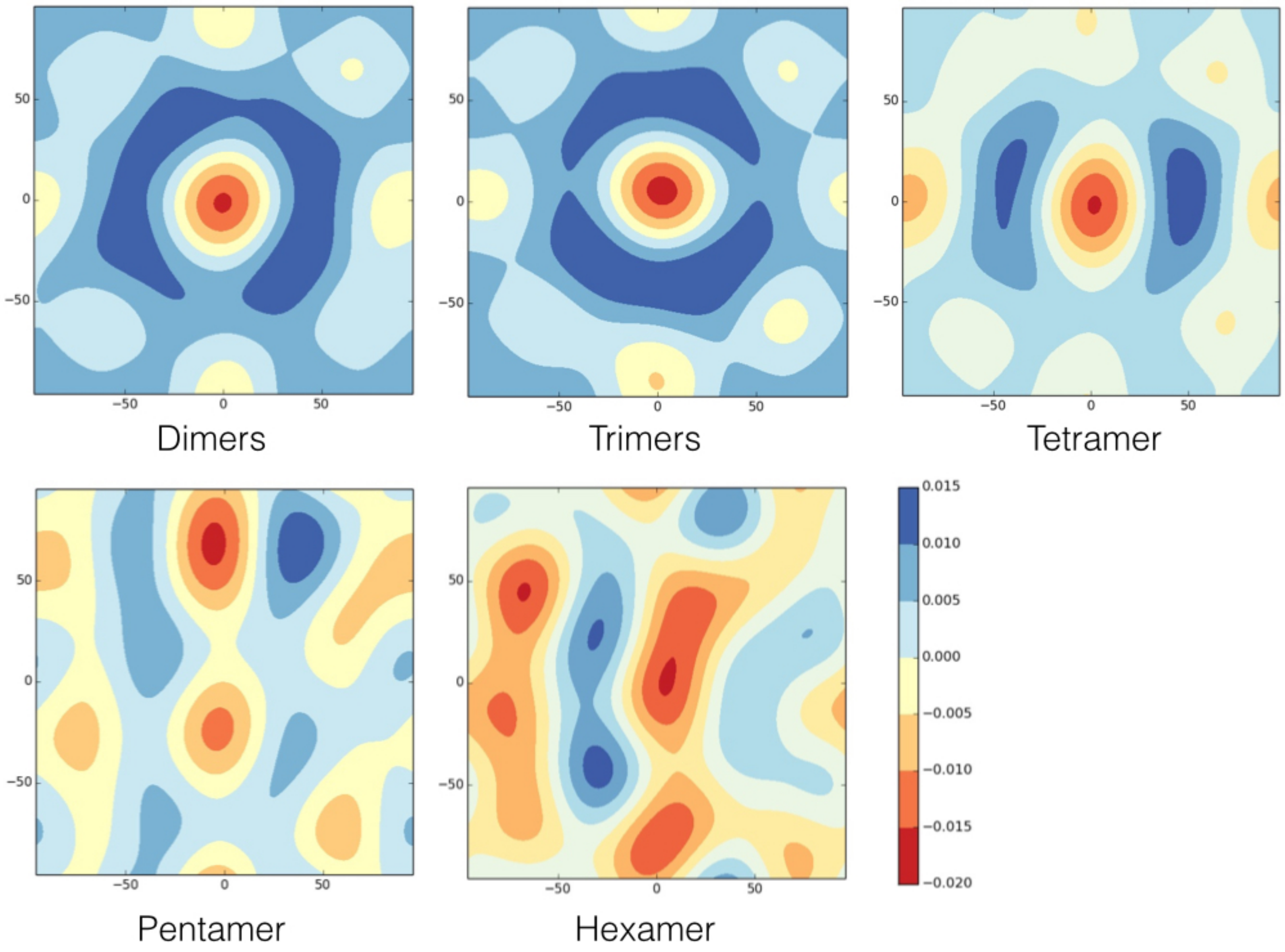

Figure 1. Heat map of the membrane curvature as induced by each type of oligomeric state for the first replica. Membrane curvature dimension is [nm^{-1}].

1.2 2ND REPLICA

In Supplementary Figure 2 are present oligomeric states up to the tetramer only because the life time of pentamers and hexamers is less than 1 μs in this replica. Despite in Supplementary Figure 2 are present oligomeric states up to the tetramer Supplementary Figure 5 and Supplementary Figure 6 show also the curvature induced by pentamers and hexamers.

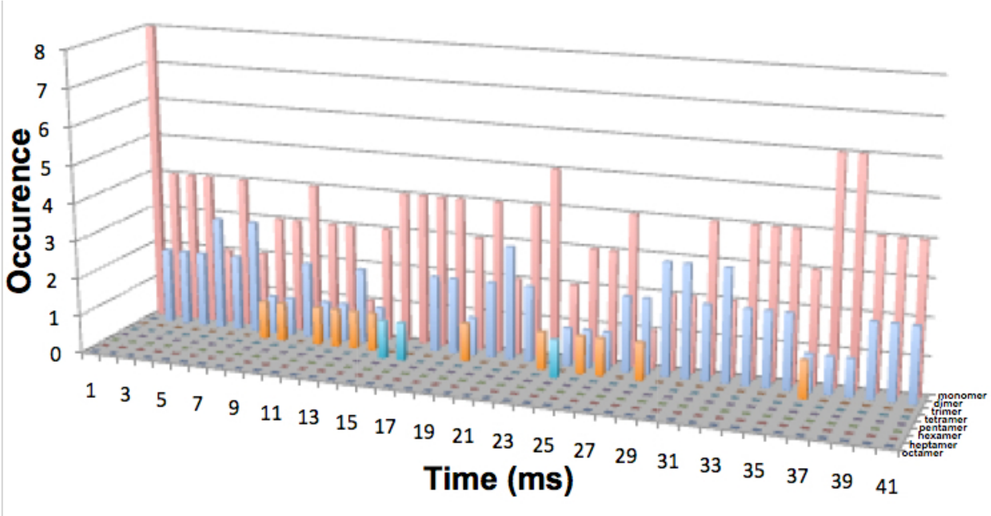

Supplementary Figure 2. Evolution of the oligomer formation during the time for every microsecond of simulation for the 2nd replica. The monomers are represented in pink, the dimers in cyan, the trimers in orange and the tetramer in blue.

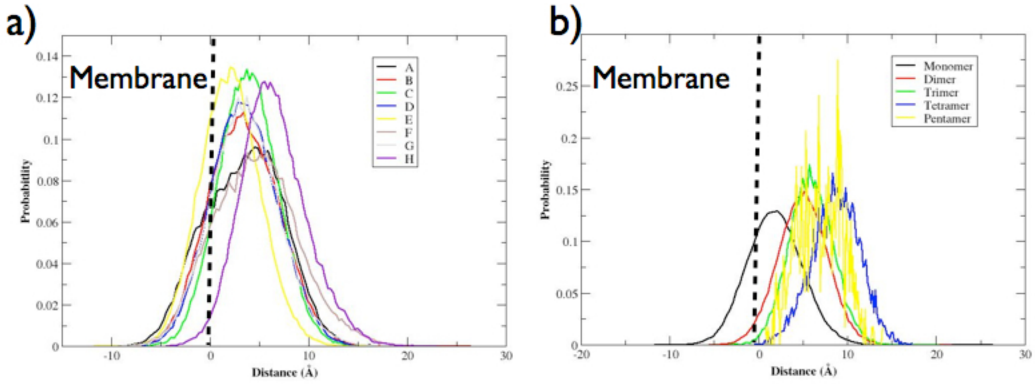

Supplementary Figure 3. Probability distribution of positioning of peptides with respect to the membrane surface for the 2nd replica. In this figure the probability distributions for the hexamers are not showed because too noisy. (a) Probability distribution as calculated for each single monomer. (b) Probability distribution as calculated for oligomeric state.

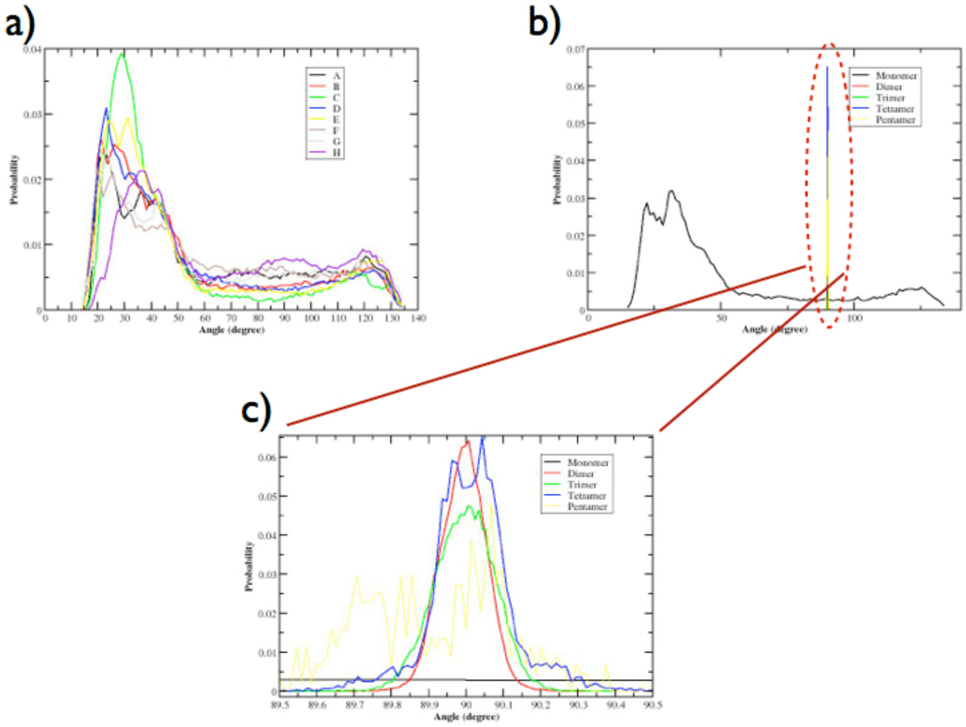

Supplementary Figure 4. Probability distributions of monomers and oligomers orientations with respect to the membrane surface for the 2nd replica. In this figure the probability distributions for the hexamers are not showed because too noisy. (a) Probability distributions as calculated for each single monomer. (b) Probability distributions as calculated for oligomeric state. (c) Zoom of the probability distributions as calculated for oligomeric states from dimers up to pentamer.

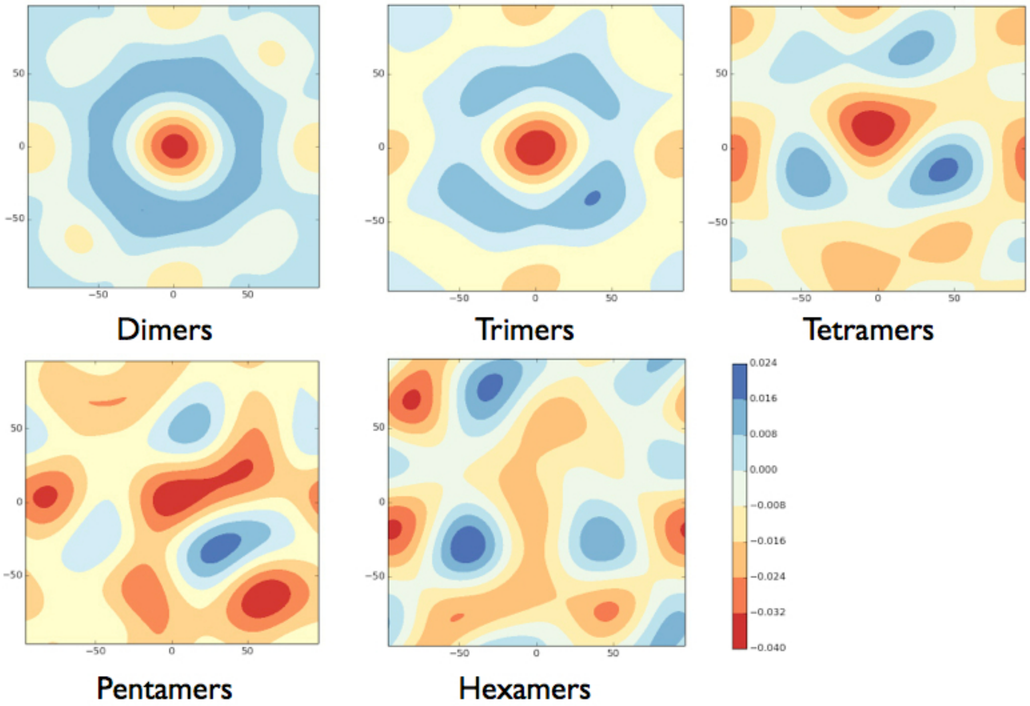

Supplementary Figure 5. Heat map of the membrane curvature as induced by each type of oligomeric state for the 2nd replica. Membrane curvature dimension is [nm^{-1}].

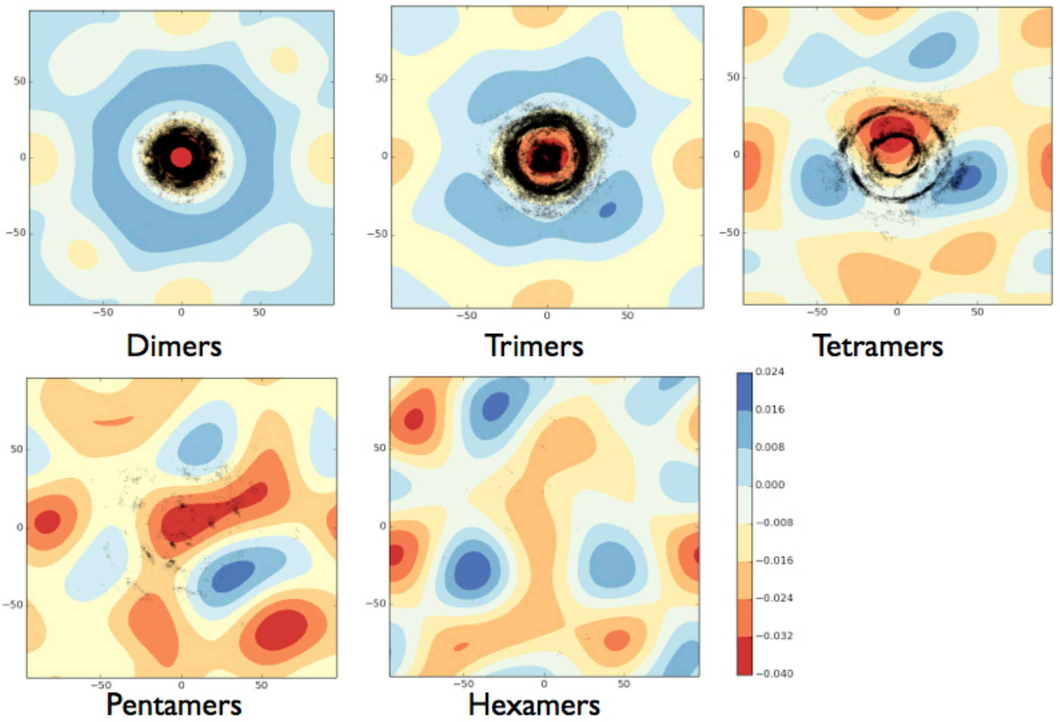

Supplementary Figure 6. Heat map of the membrane curvature as induced by each type of oligomeric state for the 2nd replica. Black dots correspond to the center of mass of each monomer belonging to the oligomer. Membrane curvature dimension is [nm^{-1}].

1.3 3RD REPLICA

In Supplementary Figure 7 are present up oligomeric states up to the tetramer only because the life time of pentamers is less than 1 μs in this replica and hexamers were not found in the trajectory. Supplementary Figure 10 and Supplementary Figure 11 also show the curvature induced by pentamers.

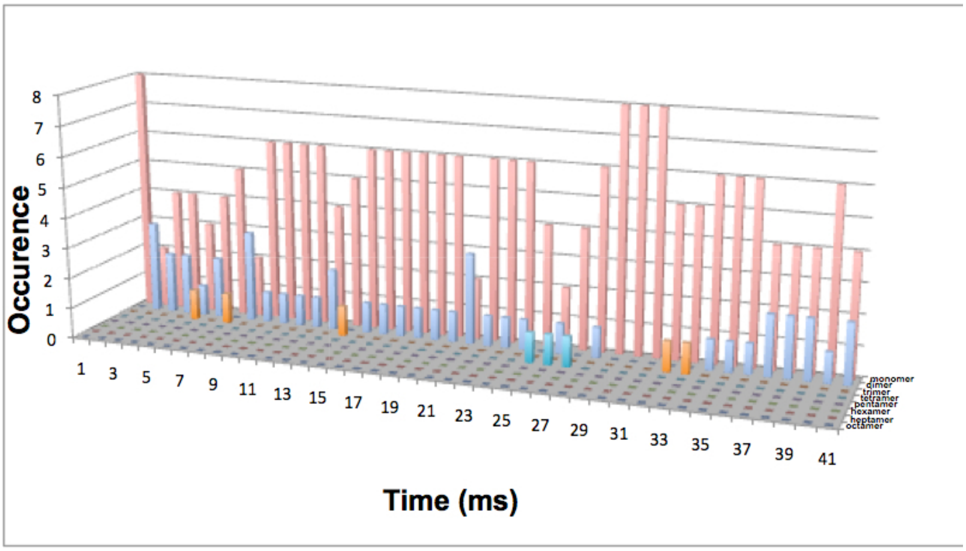

Supplementary Figure 7. Evolution of the oligomer formation during the time for every microsecond of simulation for the 3rd replica. The monomers are represented in pink, the dimers in cyan, the trimers in orange and the tetramer in blue.

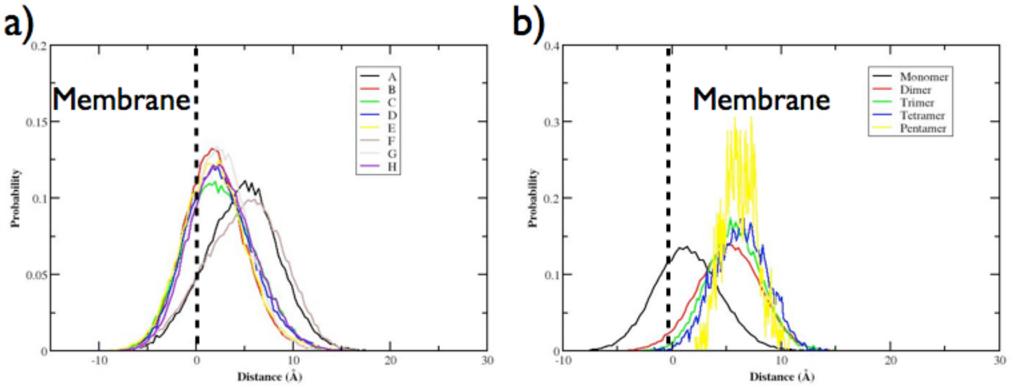

Supplementary Figure 8. Probability distribution of positioning of peptides with respect to the membrane surface for the 3rd replica. (a) Probability distribution as calculated for each single monomer. (b) Probability distribution as calculated for oligomeric state.

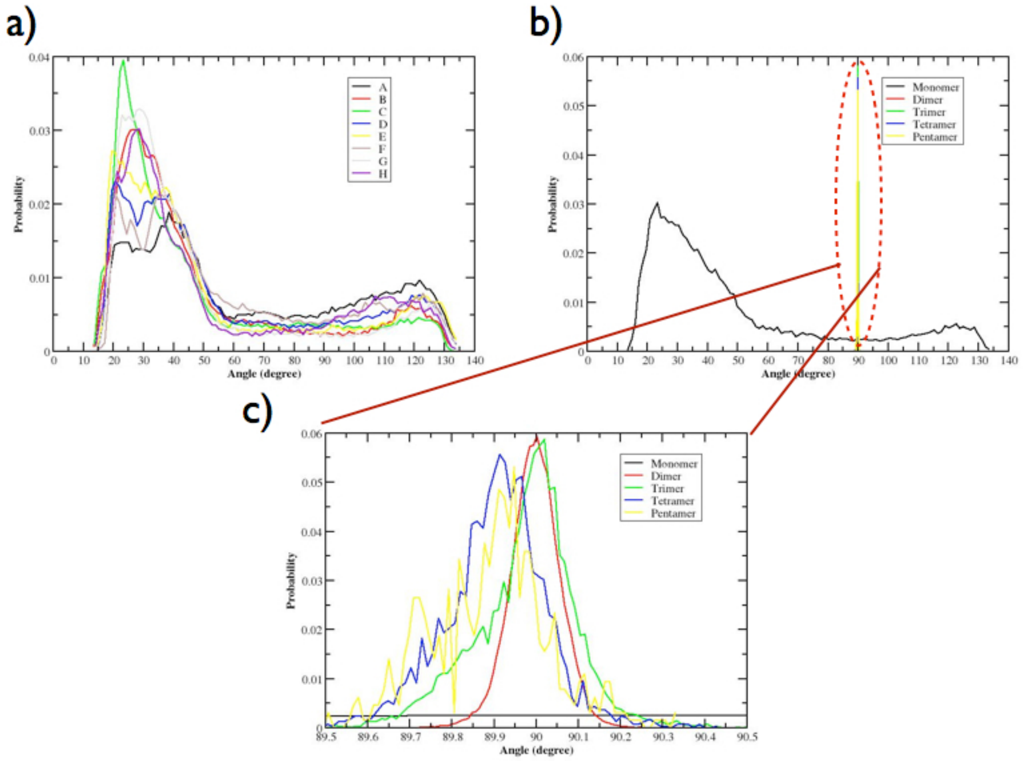

Supplementary Figure 9. Probability distributions of monomers and oligomers orientations with respect to the membrane surface for the 3rd replica. (a) Probability distributions as calculated for each single monomer. (b) Probability distributions as calculated for oligomeric state. (c) Zoom of the probability distributions as calculated for oligomeric states from dimers up to pentamer.

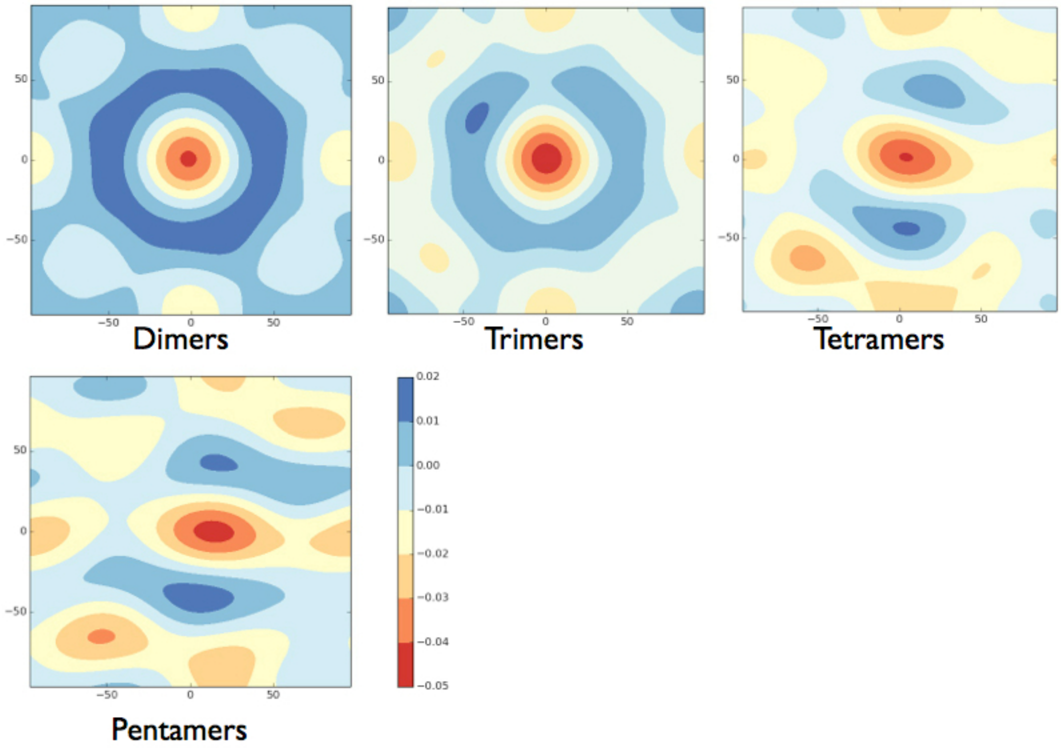

Supplementary Figure 10. Heat map of the membrane curvature as induced by each type of oligomeric state for the 3rd replica. Membrane curvature dimension is [nm^{-1}].

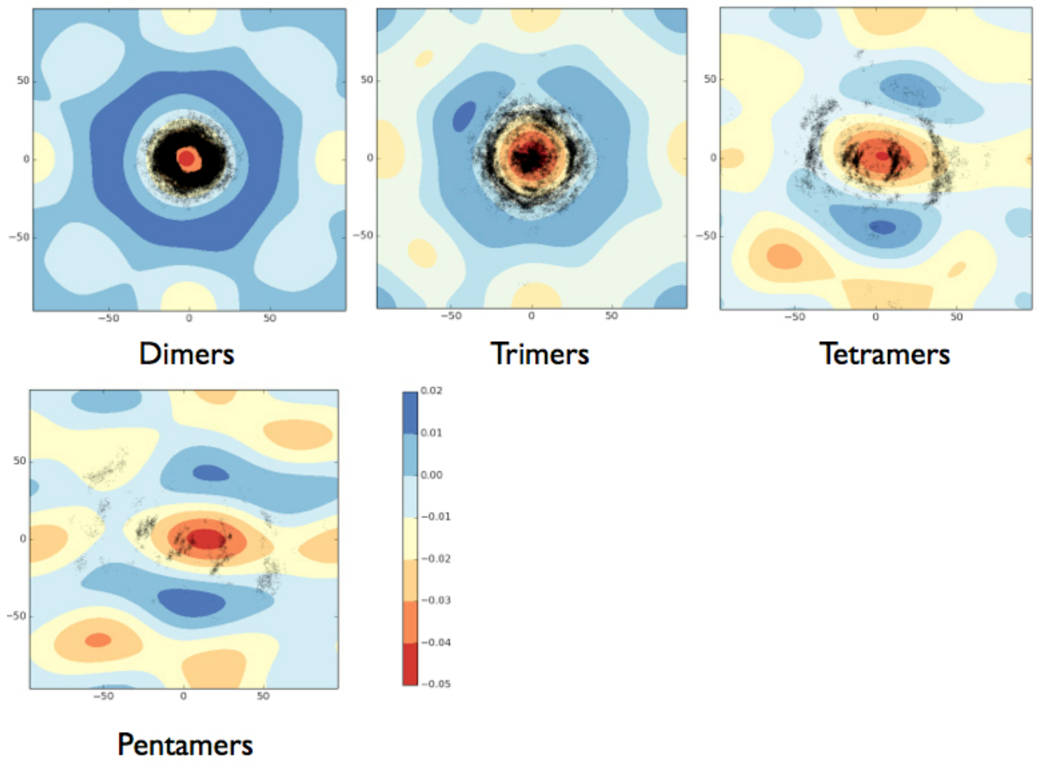

Supplementary Figure 11. Heat map of the membrane curvature as induced by each type of oligomeric state for the 3rd replica. Black dots correspond to the center of mass of each monomer belonging to the oligomer. Membrane curvature dimension is $[nm^{-1}]$.

1.4 4TH REPLICA

In Supplementary Figure 12 are present up oligomeric states up to the pentamer and hexamers were not found in the trajectory. Supplementary Figure 15 and Supplementary Figure 16 also show the curvature induced by pentamers.

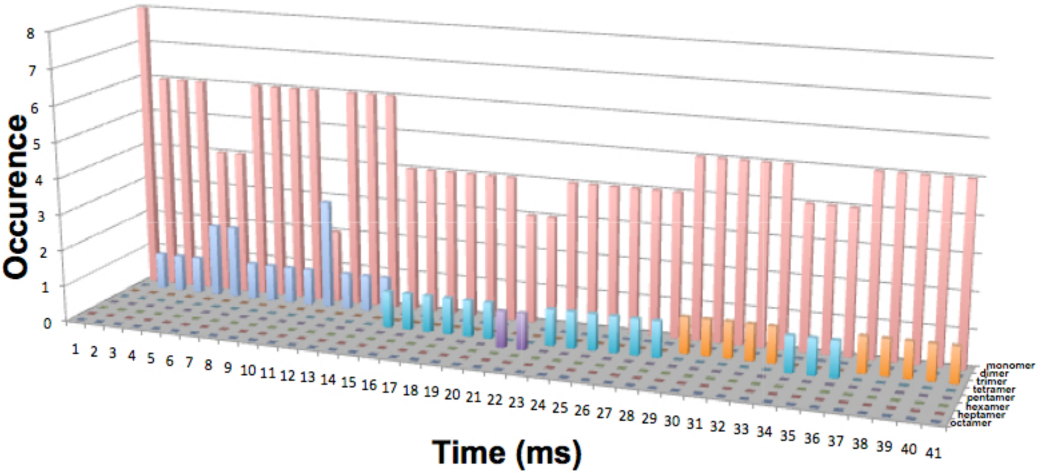

Supplementary Figure 12. Evolution of the oligomer formation during the time for every microsecond of simulation for the 4th replica. The monomers are represented in pink, the dimers in cyan, the trimers in orange, the tetramer in blue and the pentamer in violet.

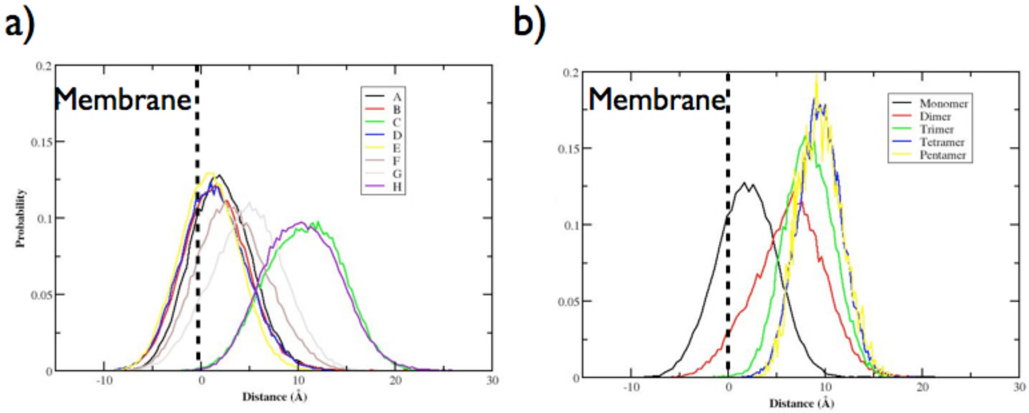

Supplementary Figure 13. Probability distribution of positioning of peptides with respect to the membrane surface for the 4th replica. (a) Probability distribution as calculated for each single monomer. (b) Probability distribution as calculated for oligomeric state.

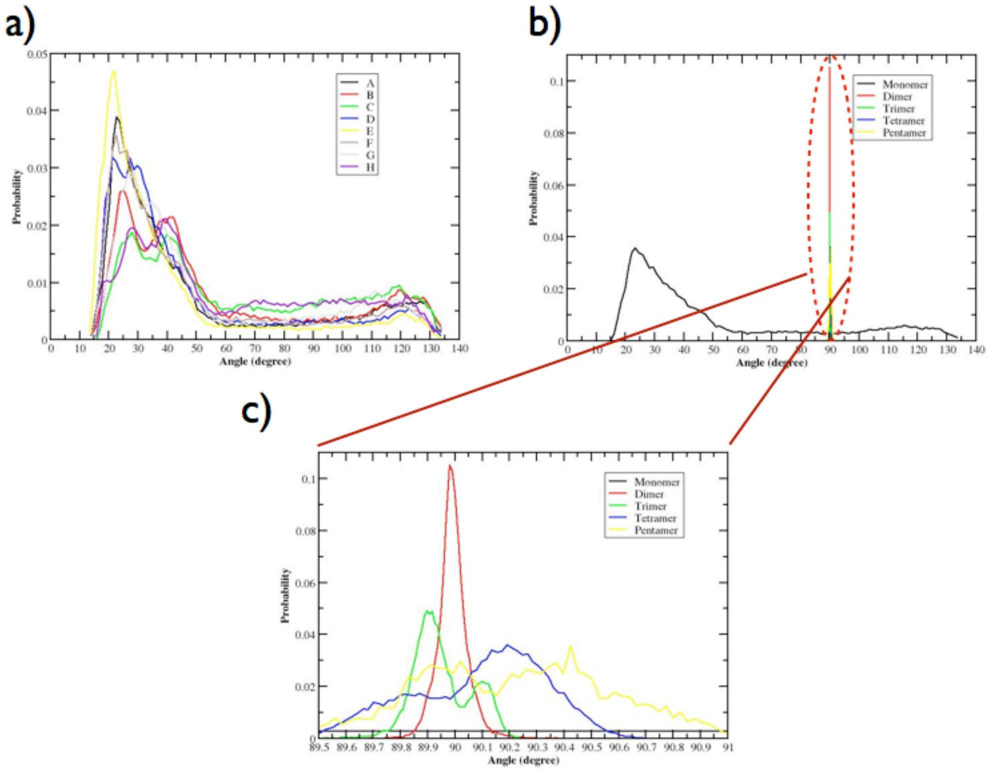

Supplementary Figure 14. Probability distributions of monomers and oligomers orientations with respect to the membrane surface for the 4th replica. (a) Probability distributions as calculated for each single monomer. (b) Probability distributions as calculated for oligomeric state. (c) Zoom of the probability distributions as calculated for oligomeric states from dimers up to pentamer.

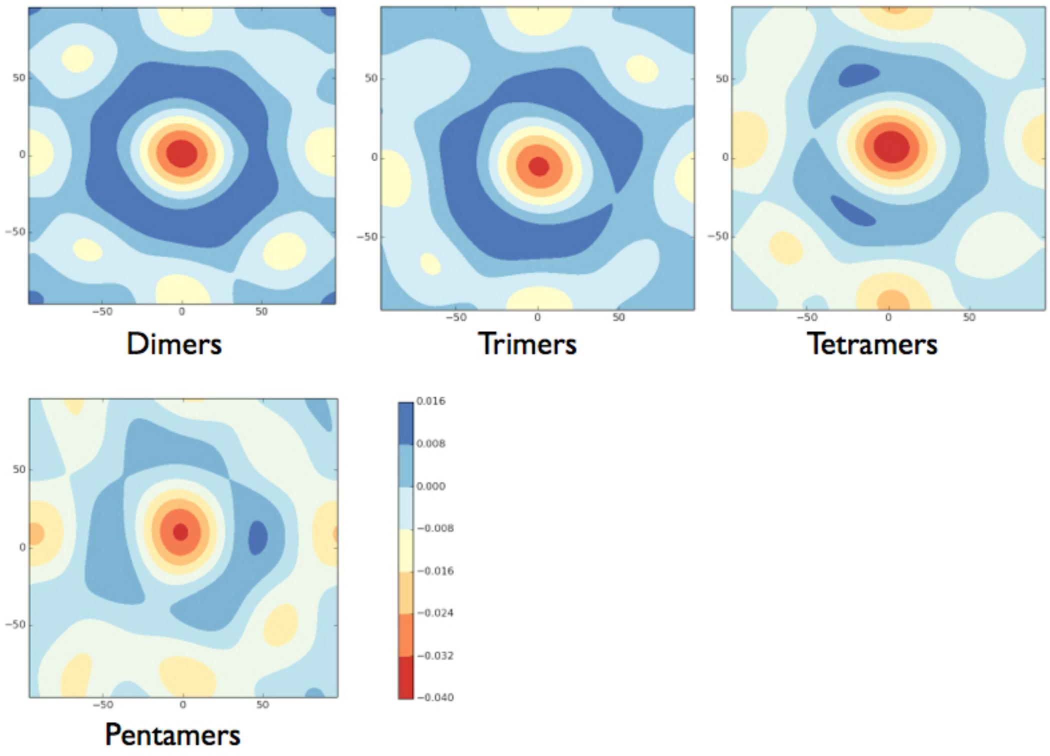

Supplementary Figure 15. Heat map of the membrane curvature as induced by each type of oligomeric state for the 4th replica. Membrane curvature dimension is [nm^{-1}].

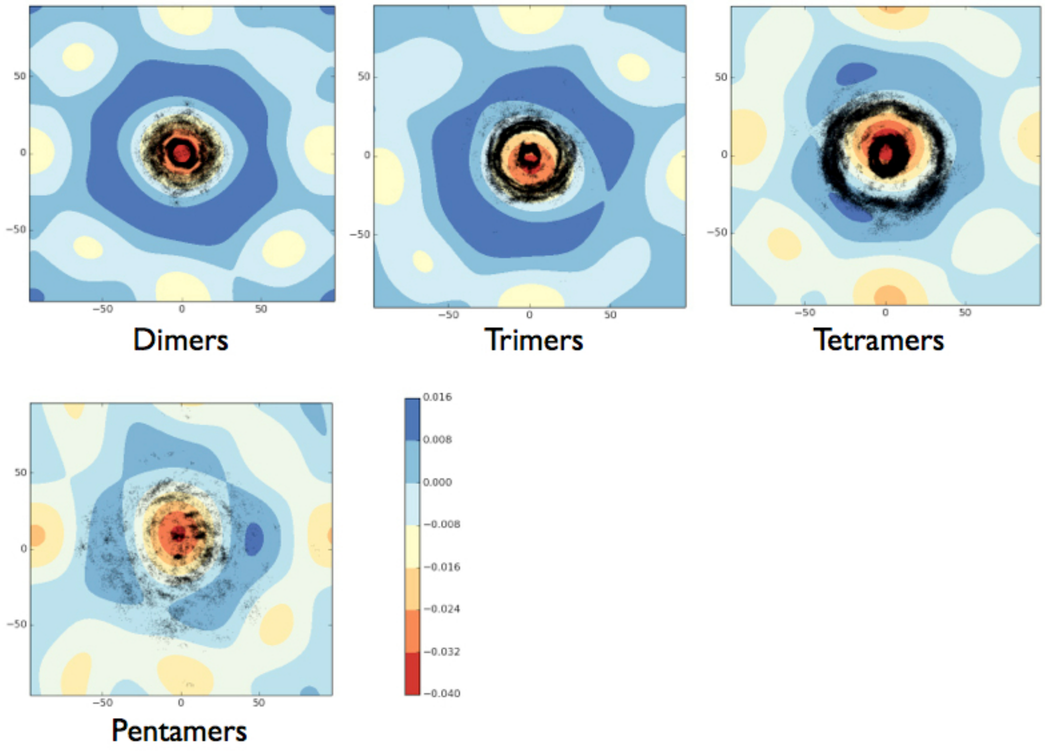

Supplementary Figure 16. Heat map of the membrane curvature as induced by each type of oligomeric state for the 4thrd replica. Black dots correspond to the center of mass of each monomer belonging to the oligomer. Membrane curvature dimension is [nm^{-1}].
